# “A fly appeared”: *sable*, a classic *Drosophila* mutation, maps to *Yippee*, a gene affecting body color, wings, and bristles

**DOI:** 10.1093/g3journal/jkac058

**Published:** 2022-03-10

**Authors:** Derek M Dean, David L Deitcher, Caleigh O Paster, Manting Xu, David W Loehlin

**Affiliations:** 1 Department of Biology, Williams College, Williamstown, MA 01267, USA; 2 Department of Neurobiology and Behavior, Cornell University, Ithaca, NY 14853, USA

**Keywords:** *Drosophila*, *sable*, body color, bristle, wing, *Yippee*, YPEL gene family, *suppressor of sable*

## Abstract

Insect body color is an easily assessed and visually engaging trait that is informative on a broad range of topics including speciation, biomaterial science, and ecdysis. Mutants of the fruit fly *Drosophila melanogaster* have been an integral part of body color research for more than a century. As a result of this long tenure, backlogs of body color mutations have remained unmapped to their genes, all while their strains have been dutifully maintained, used for recombination mapping, and part of genetics education. Stemming from a lesson plan in our undergraduate genetics class, we have mapped *sable^1^*, a dark body mutation originally described by Morgan and Bridges, to *Yippee*, a gene encoding a predicted member of the E3 ubiquitin ligase complex. Deficiency/duplication mapping, genetic rescue, DNA and cDNA sequencing, RT-qPCR, and 2 new CRISPR alleles indicated that *sable^1^* is a hypomorphic *Yippee* mutation due to an mdg4 element insertion in the *Yippee* 5′-UTR. Further analysis revealed additional *Yippee* mutant phenotypes including curved wings, ectopic/missing bristles, delayed development, and failed adult emergence. RNAi of *Yippee* in the ectoderm phenocopied *sable* body color and most other *Yippee* phenotypes. Although *Yippee* remains functionally uncharacterized, the results presented here suggest possible connections between melanin biosynthesis, copper homeostasis, and Notch/Delta signaling; in addition, they provide insight into past studies of *sable* cell nonautonomy and of the genetic modifier *suppressor of sable*.

## Introduction

Visible mutant phenotypes are central to our understanding of genetics. They allowed scientists like Mendel and Sturtevant to identify core principles of inheritance decades before DNA sequencing and transgenic technology were available, and they continue to provide easily quantifiable traits for current research. Visible mutant phenotypes in *Drosophila* further serve as simple, sensitive models of complex biological processes in developmental, molecular, and evolutionary genetics (e.g. Mullins and Rubin 1991; Wittkopp *et al.* 2003; Golovnin *et al.* 2005; Elgin and Reuter 2013; Takahashi 2013b; Dean *et al.* 2015). Mutations affecting *Drosophila* body color have been particularly and broadly informative. For example, studies of the body color genes *yellow*, *tan*, and *ebony* have improved our understanding of pigment biosynthesis, phenotypic plasticity, and rapidly evolving spot and stripe patterns within and between *Drosophila* species (Wittkopp *et al.* 2002; Gibert *et al.* 2007; Takahashi 2013a; Yamamoto and Seto 2014; Massey and Wittkopp 2016; Massey *et al.* 2019a, 2019b; Sramkoski *et al.* 2020).

**Fig. 1.**
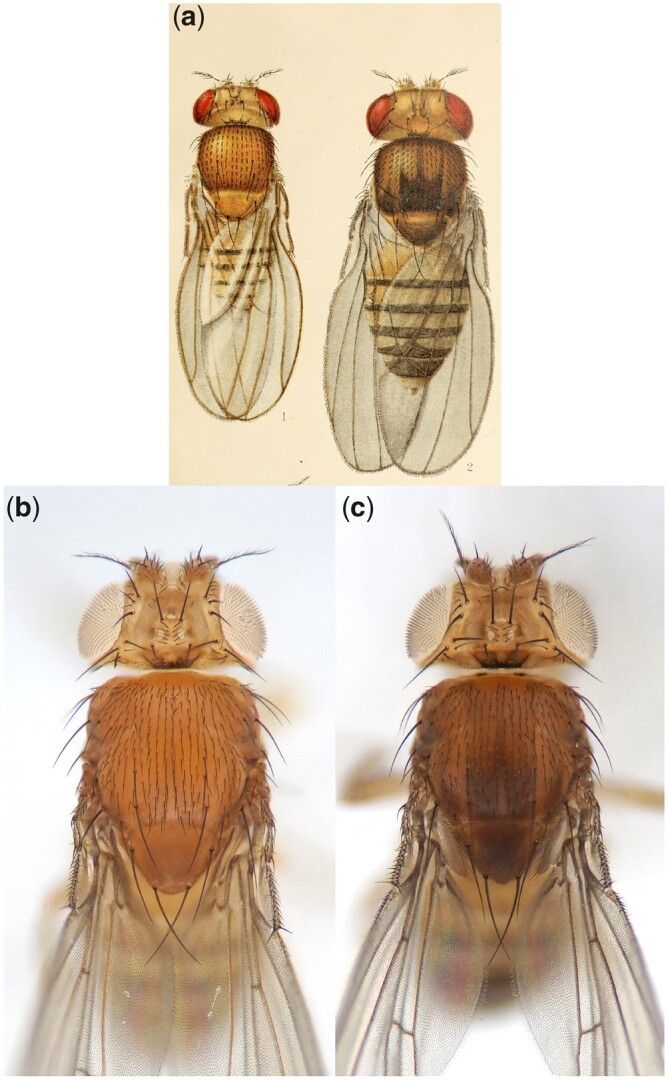
Wild-type vs *sable* body color. a) Scan from original *sable* report. Left, wild-type fly with light copper-tan body color. Right, *sable* mutant with dark brown-black body color and anteriorly pointing “trident” on its dorsal thorax. *sable* is not known to affect body size, so it is likely that the mutant was drawn relatively large to display phenotypic detail. (Images from Morgan and Bridges 1916, Plate I. Obtained from HathiTrust Digital Library, no copyright.) b) Current photo of a *sable^+^* fly (*s^+^*), showing wild-type body color. Mutant white eye color is unrelated to *sable*—as discussed in the *Materials and Methods*, we used a *white^1118^* strain for our “wild type” control stock (*w^1118^*; BL 6326). c) Current photo of a *sable^1^* fly (*s^1^*), showing the dark body color trait that we will map in this report. As in (b), eyes are white because of a *w^1118^* background. In our hands, the tridents of *s^1^* flies tended to be somewhat diffuse relative to published *s^1^* descriptions (e.g. compare c to a).

During the early decades of *Drosophila* research, a large number of viable and readily recognizable adult body color mutations were discovered and cultivated in strains. These classic mutations have been invaluable for genetic mapping and as markers for balancer chromosomes (Lindsley and Zimm 1992; Bloomington *Drosophila* Stock Center), but many have remained unmapped to their genes as research questions and tools have evolved. For example, the *sable* body color mutation, which darkens the normally copper/tan cuticle of *Drosophila* to a dark brown/black tone (Fig. 1) has intrigued geneticists since its discovery over 100 years ago, but its associated gene has not been identified. Like many other compelling findings from early genetics research, *sable* was discovered serendipitously. In the process of characterizing *black*, an autosomal body color mutation, Morgan and Bridges (1916) noticed an outlier within their *black* mutant strain. They wrote: “…a fly appeared (July 19, 1912) whose body color differed slightly from ordinary black in that the trident mark on the thorax was sharper and the color itself was brighter and clearer…the new black color, which we call sable, was due to a sex-linked factor.” Since then, *sable* has been mapped to the 11F1-12A1 bands of the X chromosome, close to the right of *IP3K2* (*wavy*; Deak *et al.* 1982). It has proven useful for mapping nearby loci and was even introduced as an exemplar of the classical genetic era in Siddhartha Mukherjee’s recent popular book *The Gene: An Intimate History* (Mukherjee 2016), but the *sable* gene itself has remained unidentified.

In our introductory genetics course, we used *sable* to teach undergraduates 3-point and deficiency/duplication mapping [formal lesson plan described in Dean *et al.* (2020), using a different mutant trait]. To develop a hypothesis for the molecular identity of the *sable* gene, students generated mapping data, then cross-referenced these data with the Flybase and GBrowse databases (McQuilton *et al.* 2012; Thurmond *et al.* 2019). The fact that *sable* was a classical mutation in an unidentified gene lent real-world intrigue to the exercise. Student enthusiasm, along with the potential to contribute to ongoing studies of *Drosophila* pigmentation-evolution, inspired us to recruit student coauthors and work together to determine the genetic basis of *sable*. Here, we report mapping, genetic manipulation, DNA sequencing, and expression studies that suggest and support the hypothesis that the *sable* phenotype results from mutations in the *Yippee* gene (*CG1989*).

## Materials and methods

### Fly stocks

The first part of the Supplementary Materials and Methods describes all the fly stocks that were used in this study: their full genotypes, providers, stock numbers, descriptions, and references (Supplementary Materials and Methods > I); in this file, stocks are grouped according to their associated experiment (Experiments 1–6). The Bloomington (BL), Vienna (V), and Zurich FlyORF (F) Stock Centers provided most of our fly lines. We also created 2 new *Yippee* mutant lines: *Yippee^Chi-A^* and *Yippee^Δ1^*; their construction is summarized in the next section of this *Materials and Methods* (*Construction of CRISPR Mutants*), with full details provided in Supplementary Materials and Methods > III.

**Fig. 2.**
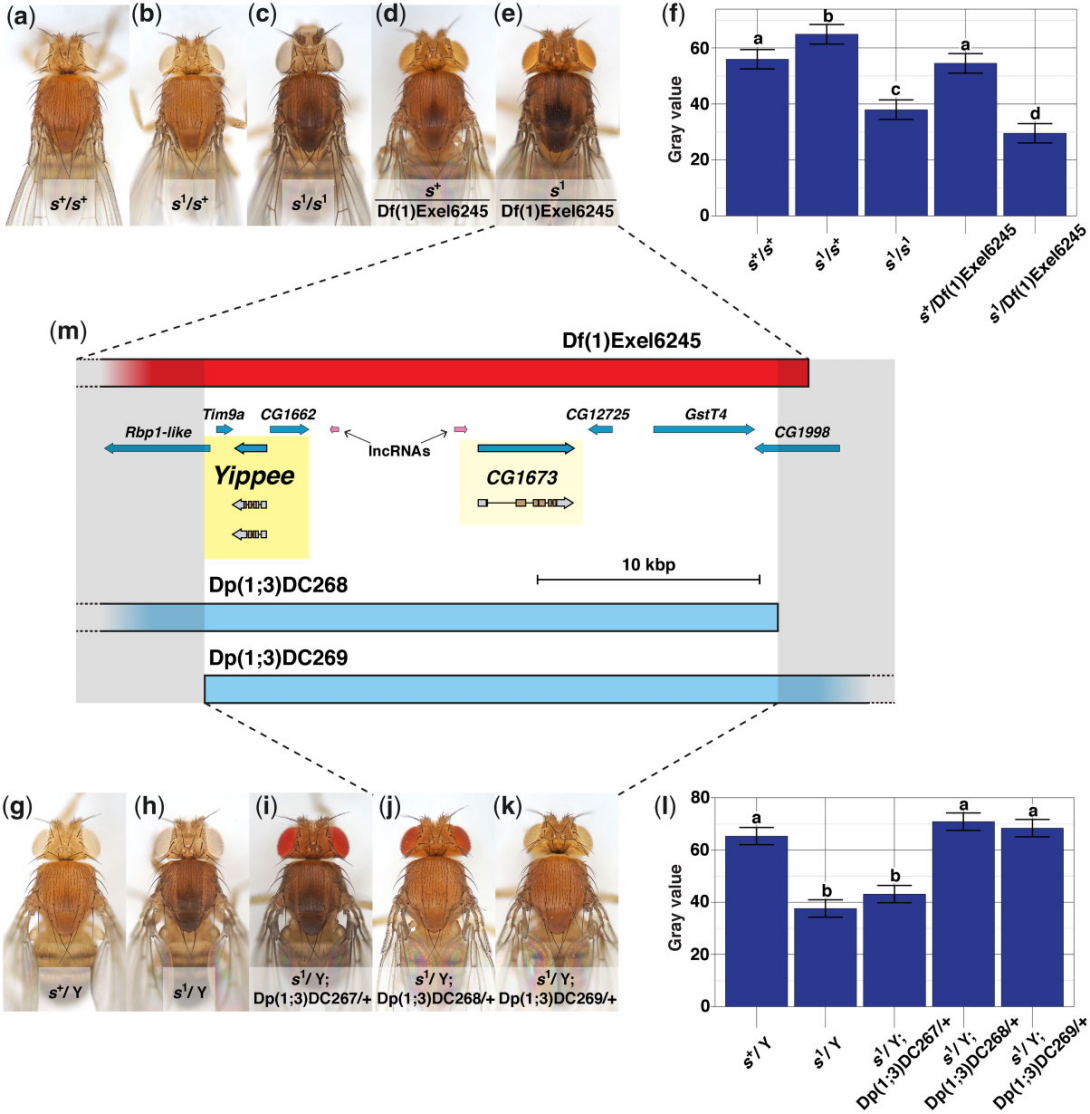
Quantification of *sable* (*s*) body color and mapping *s^1^* to a short, molecularly defined X-chromosome interval: (a–f), deficiency (Df) mapping of *s^1^*; (g–l), duplication (Dp) mapping of *s^1^*; and (m) integrates these data with GBrowse. Fly stocks and experimental cross schemes are described in Supplementary Materials and Methods > I–II > Experiment 2. a) *s^+^*/*s^+^* female from our wild-type control strain, showing copper-tan body color. b) *s^1^*/*s^+^* female as a control for the complementation test in (e). c) In contrast, an *s^1^*/*s^1^* female has a dark cuticle, particularly across the dorsal thorax. d) *s^+^*/Df(1)Exel6245 female as a second control for the complementation test in (e). e) *s^1^*/Df(1)Exel6245 female. The Df(1)Exel6245 deletion fails to complement *s^1^*, and in fact appears to enhance the phenotype relative to *s^1^*/*s^1^* (note the prominent trident). f) Quantification of body color in the deficiency mapping experiment: Least squares means plot of pixel gray values from the scuta of flies with the (a)–(e) genotypes. *s^1^*/*s^1^* and *s^1^*/Df(1)Exel6245 were significantly darker than controls, and *s^1^*/Df(1)Exel6245 were significantly darker than *s^1^*/*s^1^*. The latter observation confirms, as reported by Cramer and Roy (1980), that *s^1^* is a partial loss-of-function mutation. (*n* = 15 flies/genotype, 20 pixels sampled/scutum. Error bars indicate ±95% CI. Connecting letters above columns summarize Tukey’s HSD comparisons: If 2 groups share the same letter above their associated columns, *P ≥*0.05, and if 2 groups are labeled with different letters, *P* < 0.05. Supplementary Data contain raw data and *P*-values for every pairwise comparison.) g) *s^+^*/Y male from our control strain, showing wild-type body color. h) *s^1^*/Y male, showing *sable* body color. i) An *s^1^*/Y; Dp(1;3)DC267/+ male also shows the *sable* phenotype, but on the other hand, (j) an *s^1^*/Y; Dp(1;3)DC268/+ male, and (k) an *s^1^*/Y; Dp(1;3)DC269/+ male both exhibit wild-type body color. l) Quantification of body color in the duplication mapping experiment: Least squares means plot of pixel gray values from the scuta of flies with the (g)–(k) genotypes. *s^1^*/Y males were significantly darker than *s^+^*/Y controls, and Dp(1;3)DC268 and Dp(1;3)DC269 significantly rescued *s^1^* body color. (*n* = 15 flies/genotype, 20 pixels sampled/scutum. Error bars indicate ±95% CI. Connecting letters above columns summarize Tukey’s HSD comparisons: If 2 groups share the same letter above their associated columns, *P ≥*0.05, and if 2 groups are labeled with different letters, *P* < 0.05. Supplementary Data contain raw data and *P*-values for every pairwise comparison.) Df(1)Exel6245 did not complement *s^1^*, but Dp(1;3)DC268 and Dp(1;3)DC269 did, so the *sable* locus is expected to lie where all 3 aberrations overlap. m) Hypothesized *sable* region from a GBrowse rendering (McQuilton *et al.* 2012). Df(1)Exel6245 (top, red rectangle) and Dp(1;3)DC268 and Dp(1;3)DC269 (bottom, light blue rectangles) overlap at X:13,384,630.13,410,299 (sequence bracketed by semi-transparent gray boxes). Scale bar, 10 kbp. Light blue arrows indicate coding genes. Six coding genes are completely included within this interval: *Tim9a*, *Yippee*, *CG1662*, *CG1673*, *CG12725*, and *GstT4*. Also present are 2 long noncoding RNA loci (lncRNAs, pink arrows). Arrow orientation of each coding gene and lncRNA shows 5′-3′ transcription directionality. *Yippee* and *CG1673* are highlighted in yellow because, of the 6 coding genes in this chromosomal segment, only these 2 appear to affect adult body color (Mummery-Widmer *et al.* 2009; *Results* in this manuscript). *Yippee* and *CG1673* transcripts are delineated immediately below their associated gene; the 5′- and 3′-UTRs of each transcript (gray) flank internal coding sequence (brown), and lines connecting exons represent introns.

**Fig. 3.**
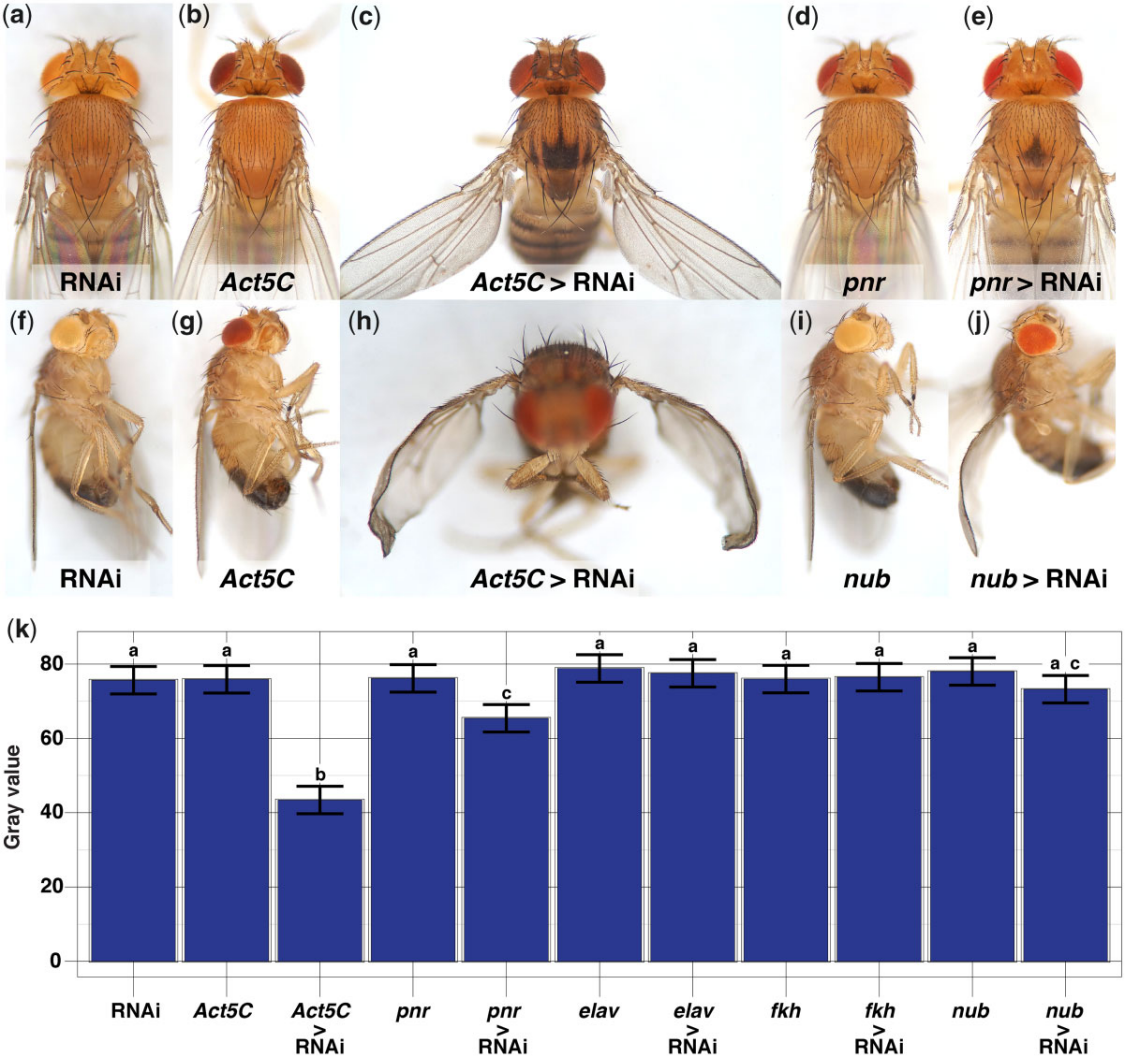
RNAi of *Yippee* can phenocopy *sable* body color (a–e, k) and can also affect wing posture and morphology (f–j). Fly stocks and experimental cross schemes are described in Supplementary Materials and Methods > I–II > Experiment 4. a) RNAi-only control, carrying one copy of the UAS-*Yippee*RNAi construct but no GAL4 driver. b) *Act5C*-GAL4-only control, with a GAL4 driver but no UAS-*Yippee*RNAi construct. Both (a) and (b) controls have wild-type copper-tan body color. c) *Act5C* > RNAi fly, showing a much darker body color than (a) and (b) controls, as well as a prominent trident on the dorsal thorax (compare to Fig. 2e). In addition, *Act5C* > RNAi flies frequently had outheld, downward-curved wings [see (h) of this figure for a clearer view]. d) A *pnr*-GAL4-only control shows wild-type copper-tan body color across its entire thorax. In contrast, (e) a *pnr* > RNAi fly has dark patches on its scutum and scutellum. f) RNAi-only control, and (g) *Act5C*-GAL4-only control, both angled to show their flat, wild-type wings. Flies in (f) and (g) also have typical wing “posture,” holding their wings dorsolaterally along the length of their bodies. h) An *Act5C* > RNAi fly as in (c), but here shown at an angle to better view the outheld posture and downward curve of the wings (quantified in Table 1). i) A *nub*-GAL4-only control has wild-type wing morphology and posture. In contrast, j) a *nub* > RNAi fly has wings that curve downwards (quantified in Table 1), but unlike *Act5C* > RNAi, its wings are not outheld. k) Quantifying effects of *Yippee*RNAi on body color: Least squares means plot of pixel gray values from the scuta. *Act5C* > RNAi and *pnr* > RNAi were the only treatments that significantly darkened scuta relative to both their RNAi-only and GAL4-only controls. (*n* = 15 flies/genotype, 20 pixels sampled/scutum. Error bars indicate ±95% CI. Connecting letters above columns summarize Tukey’s HSD comparisons: If 2 groups share the same letter above their associated columns, *P ≥*0.05, and if 2 groups are labeled with different letters, *P* < 0.05. Supplementary Data contain raw data and *P*-values for every pairwise comparison.)

In our initial experimental crosses, we mapped *sable* to a shortlist of genes using the BL 4173 *s^1^* stock (Supplementary Materials and Methods > II > Experiments 1–3). Once we had established *Yippee* as the strongest candidate, we sought to photograph and quantitatively compare phenotypes resulting from the remaining experimental crosses (Supplementary Materials and Methods > II > Experiments 4–6; also, some Experiments 2 and 3 crosses were repeated for images and quantitative data shown in Figs. 2 and 3). For this more rigorous analysis, we used stocks with overlapping genetic backgrounds: A *white^1118^* stock (*w^1118^*; BL 6326) made an appropriate “*s^+^*” control because this strain has wild-type body color, most of the stocks that we used also carried the *w^1118^* allele, and the white-eyed background facilitated the tracking of *w^+^*-marked transgenic constructs through our experimental crosses. To move *sable^1^* into a similar background, we recombined *s^1^* from BL 4173 onto the *w^1118^* X chromosome from BL 6326, then used this recombinant chromosome to establish a *w^1118^ s^1^* stock. Throughout this manuscript, we refer to *w^1118^* flies as “*sable^+^*” or “*s^+^*,” and *w^1118^ sable^1^* flies as “*sable^1^*” or “*s^1^*.”

### Construction of CRISPR mutants

*Yippee^Chi-A^* and *Yippee^Δ1^* mutants were made using CRISPR-Cas9 as described in Supplementary Materials and Methods > III. Briefly, a pair of guide RNAs was designed, with cut sites (1) in the promoter region, 28-bp upstream of the *Yippee* transcription start and (2) in the 3′ untranslated region, 62-bp downstream of the stop codon. The *Yippee^Chi-A^* mutant was produced using the CRISPaint process (Bosch *et al.* 2020), designed to insert a linearized *mini-w* construct via nonhomologous end joining (NHEJ). The *Yippee^Δ1^* mutant was produced using the homology-directed repair (HDR) CRISPR process (Gratz *et al.* 2014), designed to insert a circular *mini-w* construct containing homology arms. Marker constructs were built using MoClo modular cloning (Weber *et al.* 2011), specifically, a modified version of the MoClo Yeast Toolkit (Lee *et al.* 2015). Guide constructs were built using the KLD procedure in pU6-3-chiRNA (Gratz *et al.* 2014). Plasmid mixtures were injected into strain BL 56552 by BestGene, Inc. (Chino Hills, CA) and insertion events checked by PCR and Sanger sequencing (new allele sequences described in Supplementary Materials and Methods > IV). w *Yippee^Chi-A^*/FM7H *Bar* and w *Yippee^Δ1^*/FM7C *Bar* strains have been deposited at the Bloomington Stock Center (BL 93858 and BL 93859, respectively).

### Fly care

Flies were fed on our modified yeast/dextrose/cornmeal diet (Dean *et al.* 2015, 2020). Stocks were maintained at room temperature (19–21°C). Most of our experimental crosses were also incubated at room temperature because *sable^1^* body color is more distinguishable from *sable^+^* if flies are raised under cool conditions (Lindsley and Zimm 1992 and our observations). However, all experimental crosses involving GAL4/UAS (RNAi, misexpression, and rescue) were incubated at 25°C to increase GAL4 function, thereby increasing expression of the UAS-*Yippee*RNAi and UAS-*Yippee* constructs (Duffy, 2002).

### Experimental crosses

This manuscript often, for the sake of brevity and readability, refers to parental stocks and cross progeny by broad categories and/or standard abbreviated names. Supplementary Materials and Methods > I–II provide the information needed to fully reconstruct our experimental crosses, first by listing parental stock genotypes and sources, then by walking through the crosses that were used in this study and the genotypes of progeny that were analyzed. Crosses are subdivided into Experiments 1–6, following how we grouped their associated parental stocks. As discussed in the previous subsection (*Fly* *care*), most experimental crosses were incubated at 19–21°C (Experiments 1, 2, and 6; *CG1673* crosses in Experiment 3), but crosses involving GAL4/UAS (Experiments 4 and 5; GAL4/UAS crosses in Experiment 3) were incubated at 25°C.

### Photography and quantification of cuticle “darkness”

**Fig. 4.**
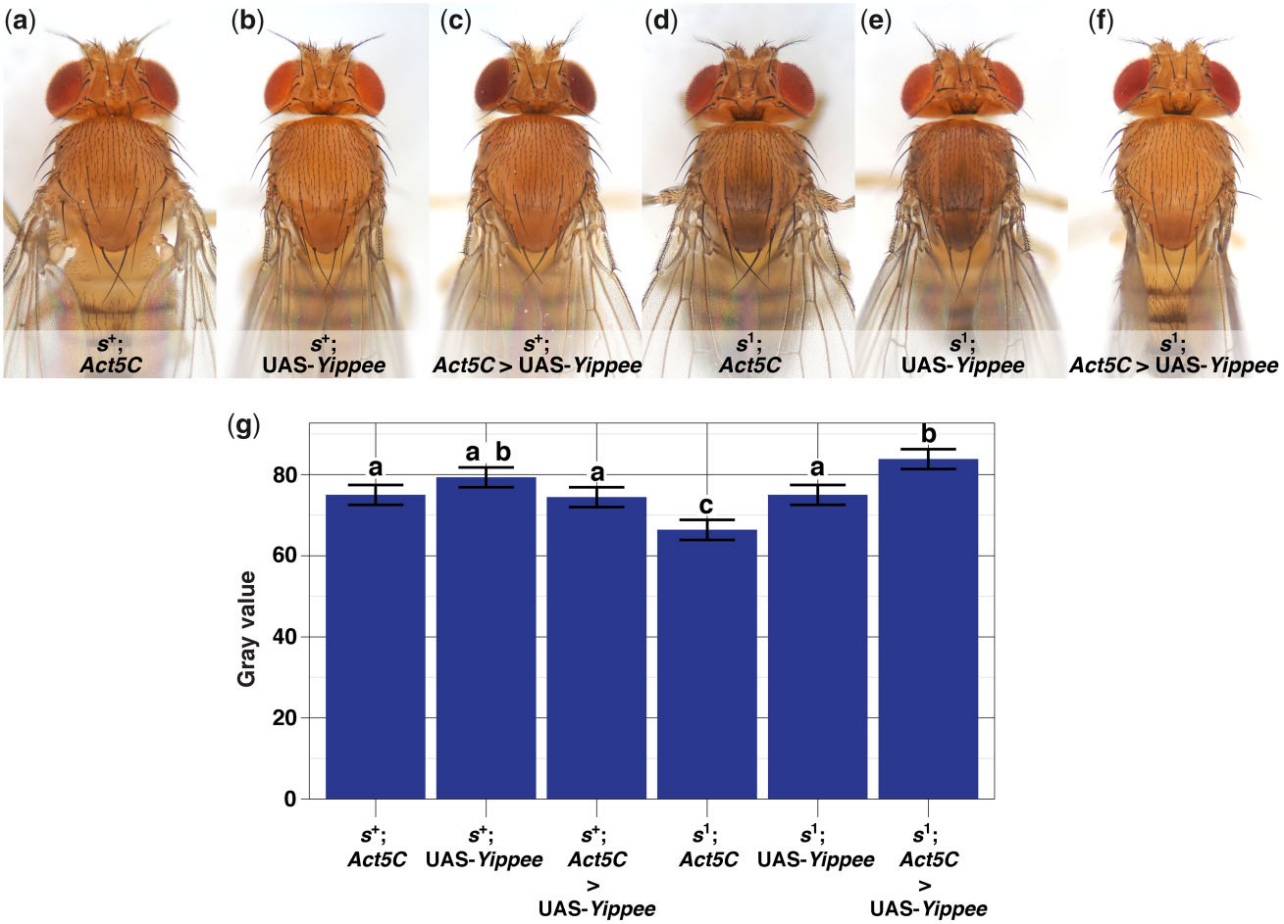
Ubiquitous misexpression of UAS-*Yippee* in *sable^+^* (a–c) and *sable^1^* flies (d–f), and quantification of their body color (g). Fly stocks and experimental cross schemes are described in Supplementary Materials and Methods > I–II > Experiment 5. a) *s^+^*; *Act5C*-GAL4-only control. b) *s^+^*; UAS-*Yippee*-only control. c) *s^+^*; *Act5C* > UAS-*Yippee*. Flies in (a)–(c) show wild-type, light body color. d) *s^1^*; *Act5C*-GAL4-only control. As expected for an *s^1^* mutant, this fly exhibits darker body color than wild-type, and a diffuse trident on the thorax. However, its body color is not as dark as that of the *s^1^* mutant flies in Figs. 1, 2, and 5—this is expected because misexpression crosses were incubated at 25°C, but other crosses involving *s^1^* were incubated at 19–21°C (see *Fly care* in *Materials and Methods* for further explanation). e) *s^1^*; UAS-*Yippee*-only control, also showing dark body color and a trident. f) *s^1^*; *Act5C* > UAS-*Yippee*. Body color is rescued to wild type, and unlike (d) and (e), no trident is visible. g) Quantifying the effects of UAS-*Yippee* misexpression on body color: Least squares means plot of pixel gray values from the scuta of flies with the (a)–(f) genotypes. *s^+^*; *Act5C* > UAS-*Yippee* gray values were not significantly different from those of *s^+^*; *Act5C* and *s^+^*; UAS-*Yippee* controls, confirming that ubiquitous misexpression of UAS-*Yippee* in an *s^+^* background is not sufficient to affect scutal color. However, UAS-*Yippee* affected *s^1^* scutal color in 2 ways: (1) Overall, *s^1^*; UAS-*Yippee* scuta were significantly lighter-colored than *s^1^*; *Act5C* scuta, even overlapping with *s^+^*; UAS-*Yippee* controls and (2) *s^1^*; *Act5C* > UAS-*Yippee* scuta were significantly lighter than *s^1^*; *Act5C* and *s^1^*; UAS-*Yippee* controls, and even lighter than *s^+^*; Act5C > UAS-*Yippee* controls. (*n* = 60 flies/genotype, 20 pixels sampled/scutum. Error bars indicate ±95% CI. Connecting letters above columns summarize Tukey’s HSD comparisons: If 2 groups share the same letter above their associated columns, *P ≥*0.05, and if 2 groups are labeled with different letters, *P* < 0.05. Supplementary Data contain raw data and *P*-values for every pairwise comparison.)

**Fig. 5.**
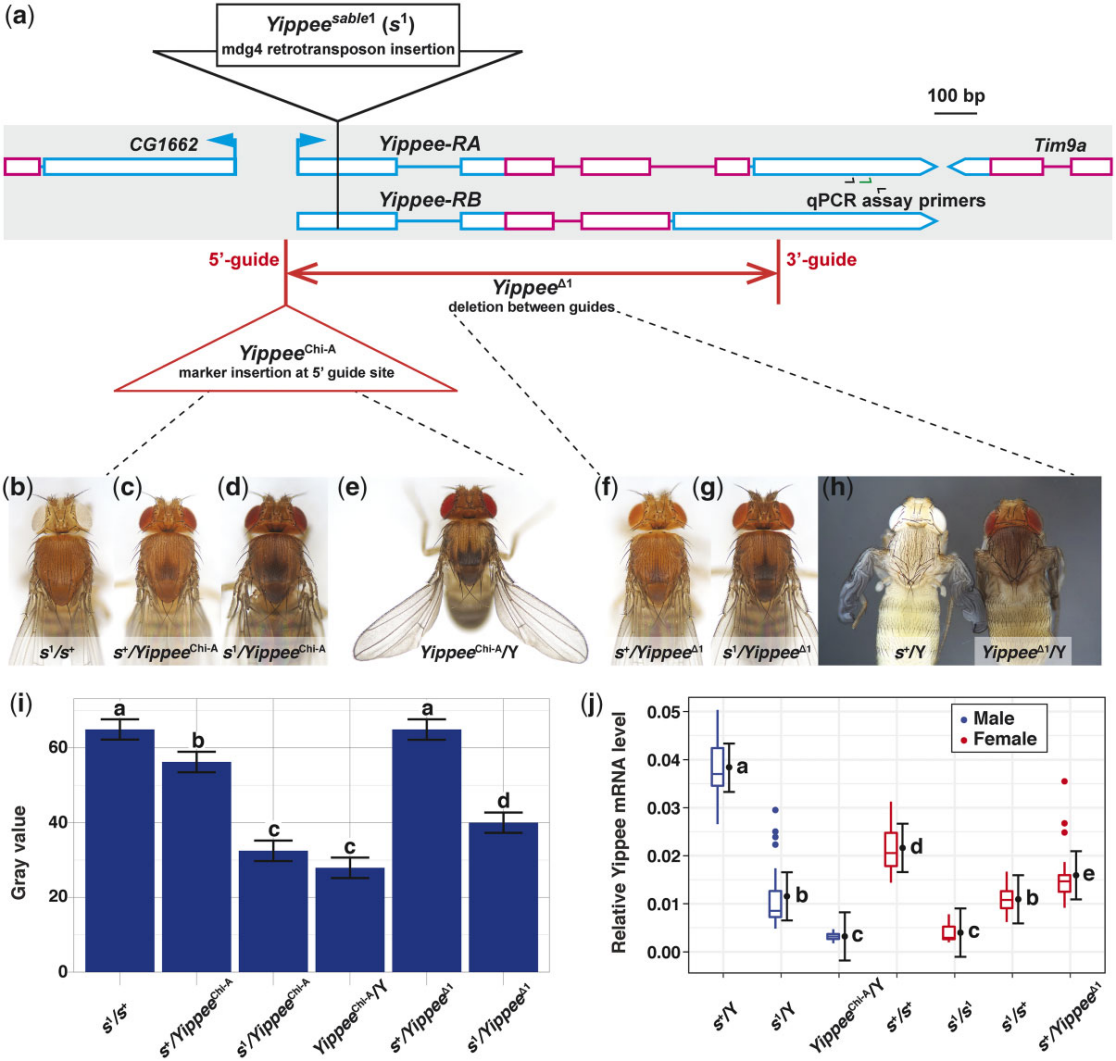
The “*Yippee^sable1^*” (*s^1^*) retrotransposon insertion and 2 new *Yippee* mutations: a) Genomic locations, b–i) mutant phenotypes and complementation tests, and j) quantification of their *Yippee* expression levels with RT-qPCR. a) *Yippee* gene region, adapted from GBrowse (McQuilton *et al*. 2012; Thurmond *et al.* 2019). Transcripts are represented by connected rectangles: noncoding segments (cyan rectangles) flank coding segments (magenta rectangles), lines between rectangles indicate introns, and arrows indicate 5′-3′ transcriptional directions. To orient the *Yippee* gene 5′-3′, sequence polarity has been switched relative to the published genome sequence (compare to Fig. 2m). A 100-bp scale bar is shown at the top right. In *sable^1^* mutants, we discovered an ∼8 kbp mdg4 element insertion in the 5′-UTR of *Yippee* (black triangle, not to scale). We also used CRISPR-Cas9 to create 2 new *Yippee* mutations (locations shown in orange): (1) *Yippee^Chi-A^*, a CRISPaint insertion 28-bp upstream of the 5′-UTR and (2) *Yippee^Δ1^*, an HDR-CRISPR deletion of the promoter, 5′-UTR, and coding regions of *Yippee*, as well as of a portion of the 3′-UTR. b–i) *Yippee^Chi-A^* and *Yippee^Δ1^* mutant phenotypes, and complementation tests with *s^1^.* Fly stocks and experimental cross schemes are described in Supplementary Materials and Methods > I–II > Experiment 6. b) *s^1^*/*s^+^* control, showing copper-tan body color. Tridents were rarely seen on flies with this genotype. c) *s^+^*/*Yippee^Chi-A^* control, also showing copper-tan body color, though a faint trident is visible. d) *s^1^*/*Yippee^Chi-A^* fly. The body is much darker than (b) and (c) controls, and a clear trident is present, indicating that the *Yippee^Chi-A^* allele failed to complement *s^1^*. e) *Yippee^Chi-A^*/Y male, showing a rather dark body, sharply delineated trident, and outheld/curved wings—note the strong resemblance to *Act5C*-GAL4 > UAS-*Yippee*RNAi flies (Fig. 3c). f) An *s^+^*/*Yippee^Δ1^* control generally exhibits copper-tan body color, but has a faint trident like the *s^+^*/*Yippee^Chi-A^* fly in (c). g) An *s^1^*/*Yippee^Δ1^* fly is much darker than the (b) and (f) controls and has a clear trident, indicating that *Yippee^Δ1^* also failed to complement *s^1^*. (H) *s^+^*/Y and *Yippee^Δ1^*/Y P13-14 pharate adults, dissected from their pupal cases. The *Yippee^Δ1^* mutant shows signs of hyperpigmenting cuticle. i) Quantifying body color: Least squares means plot of pixel gray values from the scuta of flies with the (b)–(g) genotypes. These data confirmed that (1) *Yippee^Chi-A^* and *Yippee^Δ1^* failed to complement *s^1^*, because *s^1^*/*Yippee* scuta were much darker than those of *s^1^*/*s^+^* and *s^+^*/*Yippee* controls and (2) that *Yippee^Chi-A^*/Y mutants phenocopied *sable* body color. *Yippee^Δ1^*/Y pharate adults were consistently darker colored than *s^+^*/Y pharate adults, but since they died at P13-14 and necrotic tissue rapidly darkens, we did not assess the gray values of their scuta. (*n* = 15 flies/genotype, 20 pixels sampled/scutum. Error bars indicate ±95% CI. Connecting letters above columns summarize Tukey’s HSD comparisons: If 2 groups share the same letter above their associated columns, *P ≥*0.05, and if 2 groups are labeled with different letters, *P* < 0.05. Supplementary Data contain raw data and *P*-values for every pairwise comparison.) j) Expression level of *Yippee* decreases in mutant genotypes. *Yippee* expression was determined relative to control gene *RpL32* in cDNAs of adult flies using qPCR. Tukey boxplots show the distribution of data. Error bars show 95% confidence intervals from mixed-effects model fit. Letters denote groups of Tukey HSD pairwise comparisons that are not significantly different (*P ≥* 0.05). *n* = 8 cDNAs measured per genotype, with 3 technical replicate measurements per cDNA.

Supplementary Materials and Methods > V–VII describe our figure photography workflow in detail, specifically our photography rig; how flies were collected, stored, and positioned for imaging; and how we acquired the photos that are displayed in Figs. 1–5.

For quantification of cuticle “darkness,” we followed standard recommendations from other comparative animal color studies (Stevens *et al.* 2007; Bergman and Beehner 2008; De Souza *et al.* 2017, 2020). Images for quantitative body color data were acquired and processed as described in Supplementary Materials and Methods > VIII, then data were collected, graphed, and analyzed as described in Supplementary Materials and Methods > IX. Briefly summarizing here, dorsal thoraces were photographed in RAW format. Images were imported into Adobe Photoshop CC 2015, color-corrected using an 18% gray card from the White Balance Card Set (Vello), converted to gray scale, saved as TIFFs, then imported into ImageJ 1.53i. On each fly image, 20 pixels were selected from a specific region of the scutum shown in Supplementary Materials and Methods > IX > Supplementary Fig. S5, and pixel gray values were quantified. In the RGB color scheme, gray values can range from 0 (black) to 255 (white). Therefore, dark *s^1^* mutant cuticle will tend to register lower gray value scores than brighter-colored *s^+^* controls. Complete gray value data are provided in Supplementary Data. These data were analyzed in JMP 15.1.0 and in R with package lme4 (Bates *et al.* 2015; R Core Team 2020) using mixed-effects models (replicate fly within genotype used as a nesting factor). Tukey’s HSD tests were used to make pairwise comparisons. *P*-values for all pairwise comparisons are presented in Supplementary Data. Graphs in Figs. 2–5 were made in R using packages emmeans and ggplot2 (Wickham 2016; Lenth 2021).

### Collection of bristle and wing data

Experimental cross progeny were examined under a dissecting microscope to assess scutellar bristle numbers, ocellar (OC) and postvertical (PV) bristle numbers, and wing morphology [bristle types described in Chyb and Gompel (2013); our bristle/wing data are in Table 1]. Given that *Drosophila* normally have 4 scutellar bristles, we scored a fly as having “ectopic scutellar bristles” if >4 were seen on one fly. In most cases, *Drosophila* also have 4 macrochaetes surrounding the ocelli (2 OC + 2 PV bristles), but in our experience, 3 was not an uncommon total count seen in some wild-type strains (up to 5% frequency in some lines). With this in mind, we chose to be conservative in our scoring, only counting a fly as having “missing ocellar bristles” if it had 0–2 (OC + PV) bristles total; the small proportion of flies with 3 OC bristles was considered phenotypically wild type. Wings were considered “curved” if they were noticeably bent (usually downward in the genotypes that we investigated; examples in Fig. 3). Table 1 data were imported into JMP 15.1.0, and 2-tailed, 2 × 2 Fisher’s exact tests were run to compare experimental groups vs each of their controls (comparisons described in more detail in the footnotes of Table 1).

**Table 1.** Quantification of *sable-* and *Yippee*-associated bristle and wing phenotypes.

**Genotype** ^a^	*n*	**% with ectopic scutellar bristles** ^b^	**% with missing OC/PV bristles** ^c^	**% with curved wings** ^d^
A. *sable* phenotypes, Df and Dp complementation tests				
* s^+^*/*s^+^*	100	1	0	0
* s^+^*/*s^1^*	100	4	0	0
* s^1^*/*s^1^*	100	45***	0	0
* s^+^*/Df(1)Exel6245	100	0	0	0
* s^1^*/Df(1)Exel6245	100	5	23***	0
* s^+^*/Y	100	0	0	0
* s^1^*/Y	100	22***	1	0
* s^1^*/Y; Dp(1;3)DC268/+	100	0***	0	0
* s^1^*/Y; Dp(1;3)DC269/+	100	2***	0	0
B. RNAi^e^				
* *UAS*-Yippee*RNAi	50	2	0	0
* Act5C-*GAL4	43	0	0	0
* Act5C-*GAL4 > UAS-*Yippee*RNAi	16	25*	0	82***
* pnr-*GAL4	50	14	0	0
* pnr-*GAL4 > UAS-*Yippee*RNAi	50	58***	36***	0
* nub*-GAL4	50	0	0	0
* nub*-GAL4 > UAS-*Yippee*RNAi	50	0	0	100***
C. Misexpression, Rescue				
* s^+^*/Y	100	0	0	0
* s^+^*/Y; *Act5C-*GAL4	43	0	0	0
* s^+^*/Y; UAS-*Yippee*	41	0	0	0
* s^+^*/Y; *Act5C-*GAL4 > UAS-*Yippee*	50	0	0	0
* s^1^*/Y	100	22	1	0
* s^1^*/Y; *Act5C-*GAL4	61	46	0	0
* s^1^*/Y; UAS-*Yippee*	100	6**^f^	0	0
* s^1^*/Y; *Act5C-*GAL4 > UAS-*Yippee*	96	3.1***^f^	0	0
D. New *Yippee* alleles, *Yippee*/*sable* complementation tests				
* s^+^*/*s^1^*	100	4	0	0
* Yippee* ^C*hi-A*^/*s^+^*	100	8	0	0
* Yippee^Chi-A^*/*s^1^*	100	62***	0	0
* s^+^*/Y	100	0	0	0
* Yippee^Chi-A^*/Y	60	42***	0	100***
* Yippee^Δ1^*/*s^+^*	100	0	0	0
* Yippee^Δ1^*/*s^1^*	93	25**	0	0
* s^+^*/Y, pharate adult	30	0	0	0
* Yippee^Δ1^*/Y, pharate adult	30	20*	0	0

a Supplementary Materials and Methods > I describes the full genotype of each parental stock. Experimental cross schemes are described in Supplementary Materials and Methods > II.

b Defined as >4 scutellar bristles. Examples of the ectopic scutellar bristle phenotype are shown in Fig. 3, C and E; higher resolution versions of these images are in the Supplementary Results > Supplementary Fig. S7, labeled to indicate the ectopic bristles.

c *Drosophila* normally have 2 OC bristles and 2 PV bristles, with one of these 4 bristles missing in up to 5% of cases (our observations). Here, we define a mutant phenotype as <3 (0–2) OC+PV bristles total. An example of the missing OC/PV bristle phenotype is shown in Fig. 3e; a higher resolution version of this image is in the Supplementary Results > Supplementary Fig. S7, labeled to indicate the missing bristles.

d Examples of the curved wing phenotype are shown in Fig. 3, h and j.

*Statistics:* Proportions were compared using Fisher’s exact tests (2 × 2, 2-tailed). Experimental groups were compared to each of their controls in the following configuration: (Parts A and D) Homozygous, hemizygous, and heteroallelic mutants were compared to their associated wild type and/or heterozygous controls, (Part A) each *s^1^*/Y; Dp(1;3) group was compared to the *s^1^*/Y controls, and (Parts B and C) GAL4 > UAS experimental groups were compared to their corresponding GAL4-only and UAS-only controls, as well as to controls without either transgenic construct. If an experimental group significantly differed from all of its controls, the *P*-value of the *least* significant Fisher’s exact test is indicated as follows: *P < 0.05, **P < 0.01, ***P < 10^−4^ (if no asterisk, *P* > 0.05 vs at least 1 control).

e In addition to the *Yippee*RNAi experiments listed in Part B of this table, we tested *elav* > RNAi, *fkh* > RNAi, and r4 > RNAi. None of these additional treatments affected bristles or wing morphology (*n* = 50).

f Both *s^1^*/Y; UAS-*Yippee* and *s^1^*/Y; *Act5C-*GAL4 > UAS-*Yippee* flies had significant rescue of the ectopic scutellar bristle phenotype relative to the *s^1^*/Y and *s^1^*/Y; *Act5C-*GAL4 controls. However, *s^1^*/Y; UAS-*Yippee* and *s^1^*/Y; *Act5C-*GAL4 > UAS-*Yippee* did not significantly differ from each other (*P* = 0.50).

### Structure and sequence of *Yippee*

#### PCR amplification and sequencing of the Yippee region from s^1^ mutants

First, the *Yippee* 5′-UTR and coding region were PCR-amplified and sequenced. *s^1^* and *w^1118^* genomic DNA were isolated using the squish extraction procedure (Gloor and Engels 1992; Gloor *et al.* 1993). PCR was conducted using Q5 High-Fidelity DNA Polymerase (New England Biolabs) under recommended conditions and each pairwise combination of the following forward (F) and reverse (R) primers (purchased from Integrated DNA Technologies): Yippee-3F- TCGGATTGCAAAGACCCCAA, Yippee-9F- GCGCAGAATGCAGTGACAAC, Yippee-3R- AATGCGTGGTTCCCGTTTTC, Yippee-9R- GTAGTCGCATGTGCTCCGT. PCR products were run through a 0.8% low-melt agarose gel in TAE. Bands were cut out of the gel and purified using the Monarch Gel Extraction Kit (New England Biolabs). Purified PCR products were Sanger sequenced at the Cornell University Biotechnology Resource Center (Ithaca, NY).

Subsequently, sequence of *CG1662* through the 5′-UTR of *Yippee* was obtained to identify CRISPR targets. Genomic DNA was isolated using a DNeasy kit (Qiagen). PCR was conducted using Q5 High-Fidelity DNA Polymerase with primers Yippee-region-F1 GCATCATGCGGCCCCAAACAAACAAGATTAGG and Yippee-seq-R4 CAAGCAAGGCATTATCGGTCTC. Reactions were cleaned up with exonuclease I and shrimp alkaline phosphatase (New England Biolabs), then Sanger sequenced by Genewiz, Inc. (South Plainfield, NJ) using primers Yippee-seq-F3 CTGGAGTTAGCTTAGAAAGTTATACAC, Yippee-seq-R4 CAAGCAAGGCATTATCGGTCTC, Yippee-seq-R5 CGCTGACCCTGAGCTGTG, and Yippee-seq-R6 GCGAAAAGGAAGCCCGTGC.

#### Sequencing the transposable element insertion in the 5′-UTR of Yippee

The transposable element (TE) insertion was amplified from *s*^1^ genomic DNA using LongAmp polymerase (New England Biolabs) with tailed primers gibpg7-Yippee-5′-Region-F1 gcggccgcgggaattcgattCCGGGCAGCCACGCAAGGATTGCAT and gibpg6-Yippee-5′region-R2 ccgcgaattcactagtgattGGTCAGGTGTCCGGTGTCAGGG. The ∼8 kbp PCR band was gel purified and assembled into pGem-T-Easy using HiFi Assembly Master Mix (New England Biolabs). Three clones were fully sequenced using Oxford Nanopore technology by Plasmidsaurus (Eugene, OR), then aligned to generate a consensus sequence.

#### Analysis of Yippee transcript structure

**Fig. 6.**
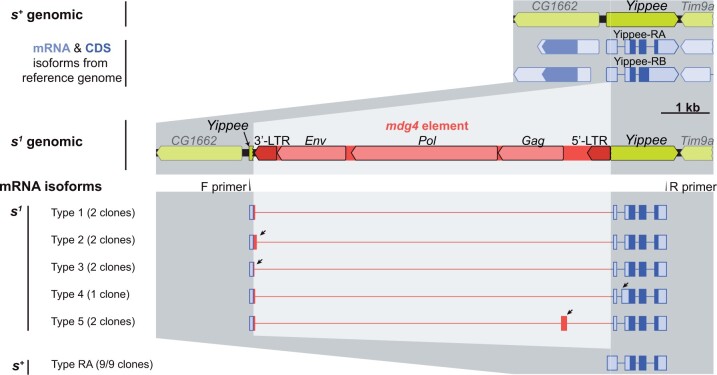
Structural organization of the *s^1^* mdg4 insertion, including *Yippee* mRNA isoforms expressed by *s^1^* mutants. mRNA was isolated from pupae, reverse transcribed, amplified using the primers shown, cloned, and sequenced. All observed *s^1^* cDNAs featured a segment of the mdg4 element spliced to the Yippee 5′-UTR, but the splice position varied. Arrowheads point to segments of *s^1^* cDNAs that differed from the Type 1 splice pattern. A similar variety of splice patterns was observed in a sample collected from *s^1^* adults (Supplementary Results > Supplementary Fig. S6).

mRNA was isolated from 12 h pupae from *w^1118^ s^+^* and *w^1118^ s^1^* strains using a Quick-RNA Tissue/Insect Kit (Zymo Research). Because the puparia are hydrophobic, we cracked them open using forceps after immersing in the lysis buffer + beads, and then homogenized the samples using a Mini-G grinder (Spex Sample Prep). cDNAs were then prepared using Superscript IV First-Strand Synthesis System with EZ DNAse (Thermo Fisher). PCR was performed using Q5 High-Fidelity DNA polymerase with 68°C annealing temperature and 4.5 min extension using primers Yippee-9F and Yippee-seq-R2 CTCCGTGGCGGATGTGC. PCR products showed multiple bands on a gel, so the remaining aliquots of PCR product were column purified (NEB Monarch kit), A-tailed, and cloned into pGem-T-Easy. Twelve individual colonies per experiment were miniprepped and inserts end-sequenced by Genewiz, Inc. A similar PCR of adult *s^1^* cDNA was performed using primers Yippee-9F and Yippee-seq-R3 GGTCAGGTTCGTGTGGCATTG, which produce a smaller product that does not contain the *Yippee* coding sequence. Pupal cDNA structures are depicted in Fig. 6 and annotated in our GenBank submission of the *s^1^ Yippee* sequence (accession number OM135585). Adult cDNAs are depicted in Supplementary Results > Supplementary Fig. S6. Sequences of pupal and adult cDNAs are provided in the Supplementary Results.

#### qPCR analysis of Yippee transcript level

For RNA isolation and cDNA synthesis, all flies were collected and frozen at −80°C before processing with the Quick-RNA Tissue/Insect kit. RNA from one fly was used per sample. mRNA was then DNAse-treated and oligo-dT-primed cDNA synthesis was performed using the Maxima H Minus First-Strand cDNA Synthesis Kit with dsDNase (Thermo Fisher). For each expression experiment, we prepared 2 sets of negative controls: (1) no-template controls and (2) -RT controls by performing all cDNA synthesis steps in the absence of reverse transcriptase; the RNA template used for the -RT control was a pool of 1 µl aliquots of the sample RNAs. No qPCR amplification was detected in no-template controls, but we did observe some qPCR amplification from most -RT controls, showing that the -RTs retained amplifiable template (e.g. off-target templates, residual genomic DNA, or sample contamination) and suggesting the various DNAse treatments were not complete. However, the observed quantification cycles for the -RT controls occurred 6 or more steps after the quantification cycle seen in the lowest expressing experimental sample, i.e. the “contaminant” concentration was less than 1/64th the lowest RNA level seen among the experimental group, suggesting a minimal effect on the quantified RNA levels.

Quantitative PCR on cDNAs was performed using multiplexed hydrolysis probe assays (Integrated DNA Technologies). *Yippee* was detected using primers qYip-F4 GTCATGAGGCTGAAGTGCTAAA, qYip-R4 GTCATGAGGCTGAAGTGCTAAA, and probe qYip-probe2-FAM/56-FAM/AAAGATGGG/ZEN/CTGCTACTCAGCTGG/3IABkFQ/. Control gene *RpL32* was detected using primers RpL-3F CAAGGGTATCGACAACAGAGTG, RpL-3R TGCACCAGGAACTTCTTGAAT, and probe Rpl32_2_probe-HEX/5HEX/TCTGATGCC/ZEN/CAACATCGGTTACGG/3IABkFQ/. Assay mix was prepared using PrimeTime Gene Expression Master Mix (IDT) according to the manufacturer’s recipe and analyzed using the PrimeTime recommended cycling conditions on a CFX96 device (Bio-Rad). A serial dilution of template tested with the multiplex assay showed amplification efficiency of >93% for each target, suggesting effective amplification in multiplex (Bustin *et al.* 2009). We also tested a number of other *Yippee* primer sets in probe and SYBR-green assays and observed similar expression results as the ones reported here. Assays were performed using matched samples on the same plate, with 3 technical replicates per cDNA sample plus -RT and no-template controls. Quantification cycle (Cq) was determined automatically by the CFX software and manually checked. For data analysis, the response variable used was expression level of *Yippee* relative to *RpL32*, computed out of the logarithmic Cq using the formula 2^Cq_Yippee^/2^Cq_RpL32^. Multiple comparisons were performed using R package emmeans on a mixed-effects model (package lme4, Bates *et al.* 2015) that included technical replicate as a random effect.

## Results

### Recombination, deficiency, and duplication mapping

Previous studies have mapped *sable* on the X chromosome, close to the right of *IP3K2* (map position 1–42) and to the left of *upheld* (1–44) and *garnet* (1–45; Morgan and Bridges 1916; Fahmy and Fahmy 1959; Deak *et al.* 1982; Larkin *et al.* 2021). We used recombination mapping between *white* (*w*), *IP3K2^wy2^* (*wy^2^*), *garnet^2^* (*g^2^*), *forked* (*f*), and *sable^1^* (*s^1^*) to verify the relative position of the *sable* locus (Supplementary Materials and Methods > I–II > Experiments 1–2). With respect to the *wy* and *s* markers, only parental type F2 males were seen (*wy s^+^* and *wy^+^ s*), i.e. no *wy s* or *wy^+^ s^+^* recombinant F2 males were found (n = 554). From these data, *w^+^* F2 males were considered for 3-point mapping of *s^1^*, *wy^2^*, and *g^2^*. This analysis showed a 0.8 cM recombination distance between *g^2^* and *s^1^* and between *g^2^* and *wy^2^*, with again no *wy s* or *wy^+^ s^+^* recombinants (n = 256; *w* flies were excluded from this part of the analysis because white eyes would have masked if flies were *g^+^* or *g^2^*). Although our results indicated a smaller *sable*—*garnet* map distance than had been reported and did not confirm which side of *IP3K2* that *sable* is on, they did confirm that *sable* is closer to *IP3K2* than it is to *garnet*. Therefore, since *IP3K2* maps to the left of *garnet*, so must *sable*.

To further narrow down the location of *sable*, we investigated whether various molecularly defined deficiencies and duplications of the region surrounding *IP3K2* could complement *s^1^*. For deficiency mapping, *s^1^*/Df(1) females were produced and scored for body color. Each type of “Df(1)” was an X chromosome with a distinct segment deleted of known sequence (Parks *et al.* 2004). If a deficiency were to remove the *sable* locus, it should not complement *s^1^* because there is no functioning copy of *sable* on its chromosome. On the other hand, a deficiency removing a region that did not include the *sable* gene should complement *s^1^* because, elsewhere on the same chromosome, an *s^+^* allele should be present. In our deficiency mapping experiments, only *s^1^*/Df(1)Exel6245 females had dark bodies, while all other *s^1^*/Df(1) females had wild-type body color (Fig. 2, a–f). This suggested that Df(1)Exel6245 was the only deficiency tested that had deleted the *sable* gene.

Deficiency mapping also enabled us to characterize and confirm basic *s^1^* allele genetics. Interestingly, we found that *s^1^*/*s^+^* controls had slightly lighter-colored scuta than *s^+^*/*s^+^* and *s^+^*/Df(1)Exel6245 controls. Otherwise, the *s^1^* dark body phenotype was recessive and scuta of *s^1^*/Df(1)Exel6245 flies were darker than those of *s^1^*/*s^1^* flies (Fig. 2, a–f). In parallel with this latter observation, Cramer and Roy (1980) had reported that *s^1^*/Df(1)C246 hemizygotes were darker than *s^1^*/*s^1^* homozygotes. From these results, they hypothesized that *s^1^* is a hypomorphic (i.e. partial loss of function) mutation. Our results using a different but overlapping deficiency were consistent with this hypothesis.

For duplication mapping, *s^1^*/Y; Dp(1;3)/+ males were produced and scored for body color. Each “Dp(1;3)” was a duplication of a distinct, wild-type segment of the X chromosome onto chromosome 3 (Venken *et al.* 2010). Duplications that span the *sable* locus should carry a functioning copy of *sable* (*s^+^*) and so would be expected to complement *s^1^*. However, duplications that do not span the *sable* gene would not be expected to complement *s^1^* because they do not carry a *sable* gene at all. Of the duplications tested, only Dp(1;3)DC268 and Dp(1;3)DC269, 2 overlapping duplications, rescued the body color of *s^1^*/Y males (Fig. 2, g–l). This suggested that the *sable* gene lies within the overlap between these 2 duplications.

### Initial screening of *sable* candidate genes

Examination of the overlap between Df(1)Exel6245, Dp(1;3)DC268, and Dp(1;3)DC269 using the Flybase GBrowse tool revealed 6 protein-coding genes fully within the region: *Tim9a*, *Yippee*, *CG1662*, *CG1673*, *CG12725*, and *GstT4* (Fig. 2m). We reviewed published phenotypic data from a genome-wide RNAi screen that used the dorsal ectoderm-specific driver *pnr*-GAL4 (Mummery-Widmer *et al.* 2009); also see IMBA database https://bristlescreen.imba.oeaw.ac.at). In this study, *pnr* > RNAi of *Tim9a*, *CG1662*, *CG12725*, and *GstT4* did not affect body color; *pnr* > *CG1673*RNAi lightened body color along the dorsal midline of the thorax; and *pnr* > *Yippee*RNAi darkened body color within the same region, but in smaller patches than those affected by *CG1673*RNAi. Concurrently, we performed similar *pnr* > RNAi experiments for 5 of the 6 genes: *Tim9a*, *Yippee*, *CG1662*, *CG1673*, and *GstT4* (Supplementary Materials and Methods > I–II > Experiment 3). Our results were entirely consistent with those reported previously: *pnr* > RNAi of *Tim9a*, *CG1662*, and *GstT4* did not affect cuticle color noticeably. *pnr* > *CG1673*RNAi caused a subtle lightening of a broad stripe along the midline of the scutellum and scutum. Finally, *pnr* > *Yippee*RNAi using either *Yippee*RNAi construct that we tested (V 46977 or V 39899) darkened small patches along the midline of the scutellum and posterior scutum (Fig. 3, a, d, e, and k).

Focusing on the 2 strongest *sable* candidates—*CG1673* and *Yippee*—we repeated the RNAi experiments with *Act5C*-GAL4, a more ubiquitous GAL4 driver than *pnr*-GAL4. Effects were qualitatively similar to *pnr* > RNAi, but stronger and wider-ranging: *Act5C* > *CG1673*RNAi generally lightened cuticle color (not shown), and *Act5C* > *Yippee*RNAi darkened broad but discrete patches of cuticle across the body (Fig. 3, a–c and k); for example, a prominent “trident” was observed on the dorsal thorax (Fig. 3, a–c), as had been reported of previous *s^1^* stocks and of *sable^ebonized^* (*s^eb^*) flies (Morgan and Bridges 1916; Morgan *et al.* 1925; Fahmy and Fahmy 1959; Lindsley and Zimm 1992). *Act5C* > *Yippee*RNAi also revealed 3 additional phenotypes: (1) The wings of most *Act5C* > *Yippee*RNAi flies were held out laterally and curved downward (Fig. 3, a–c and f–h; Table 1), a phenotype that has been reported of *s^eb^* flies (Fahmy and Fahmy 1959). (2) *Act5C* > *Yippee*RNAi flies had delayed development, reaching adulthood roughly 3–4 days later than its controls. (3) Many *Act5C* > *Yippee*RNAi flies appeared to have difficulty emerging, breaking the operculum but then becoming stuck and dying as they crawled out of the pupal case.

If either *CG1673* or *Yippee* were the *sable* gene, we would expect independent loss-of-function mutations of these genes to show dark body color in complementation tests with *s^1^*. At the start of this project, no *Yippee* or *sable* mutations other than *s^1^* were available to corroborate our findings, but we investigated 2 mutations of *CG1673*, *CG1673^EY20842^* and *CG1673^EP1023^*, which are independent TE insertion mutations in the 5′-UTR of *CG1673*. The *CG1673^EY20842^* and *CG1673^EP1023^* strains appeared to have normal body color. Through experimental crosses (Supplementary Materials and Methods > I–II > Experiment 3), we found that *CG1673^EY20842^*/Df(1)Exel6245 and *CG1673^EP1023^*/Df(1)Exel6245 flies had a somewhat lighter cuticle color than wild type, similar to that seen from RNAi of *CG1673* but across the entire body (note from Fig. 2f that *s^+^*/Df(1)Exel6245 controls had wild-type body color). However, *s^1^*/*CG1673^EY20842^* and *s^1^*/*CG1673^EP1023^* flies appeared to have normal body color. In other words, *s^1^*, *CG1673^EY20842^*, and *CG1673^EP1023^* all behaved like partial loss-of-function mutations: recessive/mild mutations with phenotypes that were enhanced over Df(1)Exel6245. However, if they had been loss-of-function mutations in the same gene, *s^1^*, *CG1673^EY20842^*, and *CG1673^EP1023^* flies would have had similar body color (i.e. all dark- or all light-colored), and the *CG1673* mutations would not have complemented *s^1^*. Neither of these predictions were met, arguing against *CG1673* as the *sable* gene.

### Tissue-specific RNAi of *Yippee*

In our initial screen of genes within the *sable* region, only RNAi of *Yippee* had phenocopied *sable*, and so *Yippee* was considered further. *Act5C* > *Yippee*RNAi had revealed several phenotypes, suggesting that *Yippee* may have several distinct functions, and *pnr* > *Yippee*RNAi had indicated that at least one of these functions is tissue-specific: *pnr*-GAL4 is a well-characterized dorsal ectoderm GAL4 driver (Heitzler *et al.* 1996; Calleja *et al.* 2000; Mummery-Widmer *et al.* 2009) and *pnr* > *Yippee*RNAi darkened the cuticle in patches along the dorsal midline without noticeably affecting wing curvature, developmental rate, or adult emergence (Fig. 3, a, d, e, and k). Noting that *Yippee* is expressed in several other tissues at moderate-to-high levels (Chintapalli *et al.* 2007; Brown *et al.* 2014), we tested if *Yippee* has additional tissue-specific functions: UAS-*Yippee*RNAi was misexpressed using GAL4 drivers that express in postmitotic neurons (*elav*), salivary glands (*fkh*), the prospective wing blade (*nub*), and fat body (r4). None of these manipulations significantly affected body color (Fig. 3k) or adult emergence as *Act5C* > *Yippee*RNAi had, and *elav* > *Yippee*RNAi and *fkh* > *Yippee*RNAi had no discernible effects. However, the remaining 2 experimental crosses reproduced the other *Act5C* > *Yippee*RNAi phenotypes that we had observed: *nub* > *Yippee*RNAi caused the wings to curve downwards, albeit held closer to the body than seen with *Act5C* > *Yippee*RNAi (Table 1 and compare Fig. 3, h–j), but *nub* > *Yippee*RNAi did not noticeably affect developmental rate. Conversely, r4 > *Yippee*RNAi did not affect wing morphology, but delayed development to adulthood by 2–3 days. Altogether, our RNAi experiments provided evidence for at least 3 tissue-specific functions of *Yippee*: (1) *pnr* > *Yippee*RNAi indicated that *Yippee* expression in the dorsal ectoderm affects body color, (2) *nub* > *Yippee*RNAi suggested that *Yippee* expression in the prospective wing ectoderm affects wing morphology, and (3) the delayed development of r4 > *Yippee*RNAi flies implied that *Yippee* expression in the fat body increases developmental rate.

### Misexpression of *Yippee*, rescue of *sable*

The above results were indirect evidence that *Yippee* could be the *sable* locus, and so we sought to test this hypothesis more directly. If *s^1^* is indeed a loss-of-function mutation in *Yippee*, then misexpression of a UAS-*Yippee* transgene in an *s^1^* fly should alleviate the mutant phenotype, provided that the GAL4 driver expresses in cells where *Yippee* functions and that UAS-*Yippee* misexpression does not cause adverse side effects such as lethality. With this in mind, *Act5C*-GAL4 was used to drive ubiquitous misexpression of UAS-*Yippee* in *s^+^* and *s^1^* backgrounds. Although *Act5C* > UAS-*Yippee* did not affect the body color of *s^+^* flies, it rescued the body color of *s^1^* mutants relative to associated GAL4-only and UAS-*Yippee*-only controls, particularly across the scutal area where we measured gray values (Fig. 4). In contrast to the *Act5C* > *Yippee*RNAi experiment, *s^+^*; *Act5C* > UAS-*Yippee* and *s^1^*; *Act5C* > UAS-*Yippee* flies appeared to have normal developmental rates, emergence, viability, and wing morphology.

### Construction of new *Yippee* mutant alleles and phenotypic analysis

Thus far, mapping, RNAi, and genetic rescue all supported *Yippee* as the *sable* gene. Additional *Yippee* alleles would enable us to further test this hypothesis in 2 ways: First, if *s^1^* is a loss-of-function mutation in *Yippee*, then other *Yippee* loss-of-function mutants should have a similar dark body phenotype. Second, complementation analysis between *s^1^* and *Yippee* alleles would test our hypothesis directly. If the *sable* phenotype was caused by loss of *Yippee* function, independent loss-of-function mutations of *Yippee* would be expected to not complement the *s^1^* allele, and so *s^1^/Yippee^-^* heterozygous flies would be predicted to have dark body color. On the other hand, if *s^1^* was due to mutation of a gene other than *Yippee*, flies should show wild-type body color.

Testing this hypothesis required independent *Yippee* loss-of-function alleles, but no *Yippee* mutations had been reported, the only other *sable* alleles known to us, *s^2^* (Morgan *et al.* 1925) and *s^eb^* (Fahmy and Fahmy 1959) were no longer available. With this in mind, we built new loss-of-function mutations by targeting the *Yippee* locus for deletion using CRISPR-Cas9, via guide-RNA sites that flank the *Yippee* coding sequence (Fig. 5a). Since it was unknown whether the deletions would produce a body color phenotype, we screened for integration of constructs that carry the *mini-w^+^* marker gene. To delete *Yippee* and insert the marker, we attempted 2 different experimental approaches, each of which makes use of a different DNA repair pathway. The first approach used the CRISPaint method (Schmid-Burgk *et al.* 2016; Bosch *et al.* 2020) to insert a linearized marker construct using the NHEJ pathway. The second approach used a circular marker construct containing flanking homologous sequence, for insertion by the HDR pathway (Gratz *et al.* 2014). Repair constructs for both strategies were built using MoClo (Modular Cloning; Weber *et al.*, 2011; Lee *et al.*, 2015) as part of a *Drosophila* MoClo toolkit that we are developing, described in the Supplementary Materials and Methods > III.

We first used the CRISPaint approach to attempt to delete *Yippee* and knock in a *mini-white* CRISPaint construct. One such marked allele was recovered and found to produce an incompletely dominant dark body color (Fig. 5, b–d and i). Using the Greek letter Chi to stand for “knock-in,” it was named *Yippee^Chi-A^*. However, PCR and sequence analysis of *Yippee^Chi-A^* mutants revealed that the *Yippee* locus was not actually deleted in this line as had been intended (Fig. 5a). Instead, the guide site in the 3′-UTR appeared to have been cut and repaired imperfectly (GGCCATCTACTCaatacttAGGG -> GGCCATCTACTCtaccctataAGGG), without deletion of the intervening *Yippee* coding sequence. At the 5′ guide site, the 5,600-bp *mini-white* marker construct had inserted, but also there was a deletion of 29 bp of *Yippee* sequence, removing −9 to −37 relative to the *Yippee* transcription start site. Thus, the 5′ deletion plus marker insertion could have disrupted the core promoter. Core promoters often contain motifs in the −20 to −30 interval (Vo Ngoc *et al.* 2019). The only canonical motifs we found in the *Yippee* core promoter region are downstream promoter element (DPE) motifs, RGWYV, at −25:−20 and +29:+24, and the −25:−20 DPE motif is deleted in *Yippee^Chi-A^.* In addition, upstream regulatory sequences may have been pushed away by the insertion of the 5,600-bp *mini-w* CRISPaint construct. Given that the *Yippee^Chi-A^* allele contains mutations in both the 5′ and 3′ regions, it is unclear which of these mutations is responsible for the associated mutant phenotypes described later in this subsection. Still, the most plausible explanation is that the marker construct insertion into the core promoter disrupts *Yippee* expression (for supporting evidence, see *Expression of Yippee in Mutants* below and Fig. 5j).

Because the CRISPaint approach did not result in the intended deletion of the *Yippee* locus, we pursued a second CRISPR method, using HDR to insert a *mini-w* construct in place of *Yippee*. This approach worked as intended, creating *Yippee^Δ1^*, a null allele that is a complete deletion of the *Yippee* 5′-UTR and coding region, along with nearly half of the 3′-UTR.

These new *Yippee* alleles further supported the hypothesis that *sable^1^* is an allele of the *Yippee* gene. First, *Yippee^Chi-A^* and *Yippee^Δ1^* phenocopied *sable*: *Yippee^Chi-A^*/Y males had a rather dark body, a prominent trident on the thorax, and outheld/curved wings (Fig. 5). These phenotypes were remarkably similar to *Act5C* > *Yippee*RNAi phenotypes (Fig. 3) as well as to older descriptions of *s^1^* and *s^eb^* flies (Morgan and Bridges 1916; Morgan *et al.* 1925; Fahmy and Fahmy 1959; Lindsley and Zimm 1992). Also, *Yippee^Chi-A^*/Y males, like *Act5C* > *Yippee*RNAi flies, often had difficulty emerging from the pupal case, becoming stuck and dying as they attempted to exit the operculum. No *Yippee^Chi-A^*/*Yippee^Chi-A^* adult or pharate adult females were seen. *Yippee^Δ1^* mutants had an even more severe phenotype: *Yippee^Δ1^*/Y males arrested at the P13-14 pharate adult stage, failing to initiate emergence at all. They tended to have darker cuticles than *s^+^*/Y pharate adults (Fig. 5h), but this was problematic to quantify because necrotic tissue darkens rapidly. As with *Yippee^Chi-A^*, no *Yippee^Δ1^*/*Yippee^Δ1^* adult or pharate adult females were seen.

Second, both *Yippee^Chi-A^* and *Yippee^Δ1^* failed to complement *s^1^* body color. Flies with *s^1^*/*Yippee^Chi-A^* and *s^1^*/*Yippee^Δ1^* genotypes had significantly darker bodies than heterozygous controls *s^1^*/*s^+^*, *s^+^*/*Yippee^Chi-A^*, and *s^+^*/*Yippee^Δ1^* (Fig. 5, b–d, f, g and i). As discussed at the beginning of this section, this is direct evidence that the *s^1^* body color phenotype results from a loss of *Yippee* function. *s^1^*/*Yippee^Chi-A^*, *s^1^*/*Yippee^Δ1^*, and heterozygous controls exhibited normal adult emergence and wing morphology.

### *sable* ^1^ mutants have a TE insertion in the 5′-UTR of *Yippee*

We next sought to determine how the *Yippee* gene was disrupted in the *sable^1^* mutant. Initial PCR and sequencing of segments of the *Yippee* gene found no mutations in the open reading frame (ORF), introns, or the portions of the 5′- and 3′-UTR that had been amplified (GenBank accession number # OM135585). However, PCR targeting the upstream region of the *Yippee* 5′-UTR failed to amplify from *s^1^* mutants. This suggested that *s^1^* mutants carry a structural disruption of the *Yippee* 5′-UTR.

To determine if this disruption was caused by a large insertion, we conducted long-PCR across the *Yippee* 5′-UTR. Consistent with this prediction, the *s^1^* allele produced a fragment that was ∼8 kbp longer than expected. We cloned and sequenced this fragment, revealing insertion of an mdg4 long-terminal repeat (LTR) retrotransposon (Gerasimova *et al.* 1983) into the *Yippee* 5′-UTR, in antisense orientation (Figs. 5a and 6; GenBank accession # OM135585). [mdg4 elements (Gerasimova *et al.* 1983; Bayev *et al.* 1984) have also been referred to as “gypsy” elements (Modolell et al. 1983). However, discussions of the potential offensiveness of “gypsy” in this context (Maučec 2013; Entomological Society of America 2021; Imbler 2021; Lipphardt *et al.* 2021), and Flybase rule 2.2.8 for gene names, suggest that the elements be referred to with a neutral synonym. With this in mind, we elect to use mdg4, but acknowledge the alternate term for the sake of connecting this study to the literature.] Such elements have been found to be the cause of many *Drosophila* mutations (e.g. Modolell *et al.* 1983). A BLAST search of the NCBI nucleotide database showed that this particular element was the closest match to *Drosophila* *melanogaster* mdg4 elements that carry a 109-bp deletion in the insulator/promoter region, such as GenBank accession DQ887186.1.

### Expression of *Yippee* in mutants

We next considered how the position of this mdg4 element insertion might disrupt *Yippee* expression. Possible mechanisms include structural disruption of the transcript, including altered splicing, early termination, or introducing an upstream ORF. Alternately, the insertion might reduce levels of transcript by decreasing transcription rate and/or destabilizing the transcript.

We first investigated the effects of the mdg4 element on *Yippee* transcript structure. We analyzed mRNA from *w^1118^ s^1^* and *w^1118^ s^+^* control pupae and adults using reverse transcription with PCR (RT-PCR) followed by gel electrophoresis. The *w^1118^ s^1^* RT-PCR product contained multiple bands between about 400 bp (the wild-type size) and 1,000 bp. To understand this pattern, we cloned the RT-PCR product and sequenced a number of clones. Each clone had an intact Yippee-PA ORF. Most of the mdg4 element had been spliced out, with residual mdg4 sequence remaining in the 5′-UTR of each transcript. Splicing patterns varied, but all rejoined with the *Yippee* 5′-UTR 55 nucleotides downstream of the mdg4 insertion (Fig. 6; Supplementary Results; GenBank accession # OM135585).

The insertion of a large DNA sequence into the 5′-UTR might inhibit gene expression by introducing upstream ORFs (uORF). uORFs can inhibit expression by inducing nonsense-mediated decay or inhibiting translation initiation from the “correct” ORF (Zhang *et al.* 2018). The mdg4 element’s standard *Gag*, *Pol*, and *Env* ORFs occur in antisense orientation to *Yippee*, so should not be translated. However, the antisense LTR region features several ATG start codons, including one located 33 bp into the LTR, and this is present in all splice variants excepting Type 3 (Fig. 6; Supplementary Results). Thus, uORFs occur in most *s^1^* mutant transcripts, but it remains to be determined whether these interfere with translation from the intact *Yippee* ORF.

Next, we investigated whether *s^1^* and other *Yippee* mutants express reduced levels of *Yippee* transcript. We performed quantitative real-time PCR on cDNA isolated from adult *w^1118^ s^+^* control flies and *w^1118^ s^1^*, *Yippee^Chi-A^*, and *Yippee^Δ1^* mutants. Significantly lower transcript levels were observed in all mutant genotypes relative to their associated wild-type control (Fig. 5j).

Two observations from the expression data are worth note. First, males and females differed in expression level, suggesting a role of sex-influenced regulation and/or dosage compensation. Second, *Yippee^Δ1^/s^+^* heterozygous females expressed at 73% of the wild-type *s^+^/s^+^* level, a significant reduction but greater than the expected 50% from missing an allele copy. Anomalously high expression in a null heterozygote resembles transvection, wherein regulatory elements uncoupled from a promoter can enhance expression of the other allele copy (King *et al.*, 2019). This might also be the result of unaccounted differences in genetic background.

### *Yippee* affects scutellar, OC, and PV bristle numbers

The same experimental crosses that we used to map the *sable* body color trait to *Yippee* also produced intriguing evidence that *Yippee* affects the number of macrochaetes on certain regions of the thorax and head (Table 1; Supplementary Results > Supplementary Fig. S7). First, loss of *Yippee* function appeared to increase the number of scutellar bristles (Table 1, “% with ectopic scutellar bristles” column). Ninety-nine percent of *s^+^*/*s^+^* female controls and all *s^+^*/Y male controls had 4 scutellar bristles, which is typical for wild-type *Drosophila* (Lindsley and Zimm 1992; Chyb and Gompel 2013), but 45% of *s^1^*/*s^1^* females and 22% of *s^1^*/Y males exhibited 5–6 scutellar bristles (Table 1A). This ectopic scutellar bristle trait appeared in parallel with the dark body color trait throughout our experimental crosses: For one thing, *Act5C*-GAL4 > UAS-*Yippee*RNAi, *pnr*-GAL4 > UAS-*Yippee*RNAi, *Yippee^Chi-A^*/Y, and *Yippee^Δ1^*/Y flies all phenocopied the *s^1^* ectopic scutellar bristle trait (Table 1, B and D). In addition, *s^1^* and the new *Yippee* alleles did not complement each other*—*i.e. *s^1^*/*Yippee^Chi-A^* and *s^1^*/*Yippee^Δ1^* flies had ectopic scutellar bristles at significantly higher frequencies than their heterozygous controls (Table 1D). Finally, *s^1^*/Y ectopic scutellar bristles were rescued by Dp(1;3)DC268, Dp(1;3)DC269, *Act5C*-GAL4 > UAS-*Yippee*, and even a copy of the UAS-*Yippee* transgene without a GAL4 driver (Table 1, A and C). All of these findings strongly suggested that the *sable* and *Yippee* ectopic scutellar bristle traits are due to loss of function in the same gene.

Second, we found evidence that *Yippee* affected the number of bristles on the dorsal head capsule (Table 1, “% with missing OC/PV bristles” column). On the vast majority of flies that we examined, we saw the expected 4 macrochaetes that surround the light-sensing ocelli: 2 OC bristles at the anterior side of the ocelli, and 2 PV bristles at the posterior (Lindsley and Zimm 1992; Chyb and Gompel 2013). Infrequently, we saw a fly that was missing only 1 OC or 1 PV bristle, but the frequency of this condition (up to about 5%) did not appear to vary significantly between the genotypes considered in this study, so as discussed in the *Materials and Methods*, these flies were considered “wild type” in our analysis. In contrast, 23% of *s^1^*/Df(1)Exel6245 and 36% of *pnr* > UAS-*Yippee*RNAi flies were missing 2 or more of the 4 macrochaetes surrounding their ocelli. In these flies, there was no clear pattern to which OC vs PV bristles tended to be missing: some *s^1^*/Df(1)Exel6245 and *pnr* > UAS-*Yippee*RNAi flies were missing both OC bristles only, some both PV bristles only, and some 1–2 OC as well as 1–2 PV bristles. Therefore, we collapsed all these phenotypes into one category.

Figures 2c, 3c, 3e, 4d, 5d, 5e, and 5g show flies with ectopic scutellar bristles, and the fly in Fig. 3e also is missing both PV bristles. To focus the narrative on mapping the *sable* body color trait, we did not directly indicate bristle defects on manuscript images, but we do elsewhere: Of all the examples, Fig. 3, a–e most clearly demonstrates both bristle phenotypes, and so we present it in full resolution in the Supplementary Results > Supplementary Fig. S7, marked with arrows to indicate affected bristles.

## Discussion

### *Yippee* is the *sable* gene

All our experiments supported the hypothesis that the *sable^1^* dark body phenotype is due to loss of *Yippee* function: Recombination, deficiency, and duplication mapping located *s^1^* at a chromosomal interval that includes *Yippee* and only 5 other coding genes (Fig. 2). Our own and a previous RNAi screen showed that, of these 6 candidate genes, only RNAi of *Yippee* darkened the cuticle (Fig. 3; Mummery-Widmer *et al*. 2009); IMBA Bristle Screen Database). Ubiquitous misexpression of UAS-*Yippee* fully rescued *s^1^* body color (Fig. 4). Two independent loss-of-function *Yippee* mutations phenocopied and failed to complement *s^1^* (Fig. 5). Finally, DNA sequencing of *s^1^* genomic DNA revealed an mdg4 retrotransposon insertion in the 5′-UTR of *Yippee*, which was associated with reduced *Yippee* mRNA levels and expression of *Yippee* mRNAs containing modified 5′-UTRs with variable lengths of spliced retrotransposon sequence (Figs. 5 and 6; Supplementary Results). In keeping with standard nomenclature practices, we propose renaming the *sable^1^* allele *Yippee^sable1^* or *Yippee^s1^*.

The allelic series of *Yippee^sable1^, Yippee^Chi-A^*, and *Yippee^Δ1^* along with the *Yippee*RNAi and UAS-*Yippee* constructs form a versatile toolkit to advance our understanding of how *Yippee* function affects the disparate traits of body color, wing morphology, developmental rate, bristle development, adult emergence, and viability. As a hypomorphic allele, *Yippee^sable1^* could be a sensitive gauge for genetic interaction studies because both enhancement and suppression of the body color phenotype could be detected. The more severe *Yippee^Chi-A^* and *Yippee^Δ1^* alleles could facilitate the study of mutant phenotypes not seen in *Yippee^sable1^* such as curved wings and pharate adult lethality. *Yippee^Δ1^* is a deletion of all *Yippee* coding sequences, and so it likely represents complete loss of *Yippee* function (Fig. 5a). We and others have found evidence of tissue-specific *Yippee* functions (*Results*, *Tissue-Specific RNAi of Yippee*; Fig. 3; Mummery-Widmer *et al.* 2009). The availability of *Yippee*RNAi and UAS-*Yippee* (Dietzl *et al.* 2007; Bischof *et al.* 2013) may help build on these findings and reveal additional tissue—as well as developmental stage-specific roles of *Yippee.*

### The biochemical and physiological role of Yippee remains unclear

The existing biochemical analyses of *sable* mutants and Yippee protein are fairly limited. *sable*, along with other dark-colored mutants *black*, *ebony*, and *tan*, all show decreased levels of β-alanine (Wright 1987). β-alanine is conjugated to dopamine to synthesize N-β-alanyl dopamine (NBAD), which, in turn, is a precursor in the formation of NBAD sclerotins (yellowish pigments) (True *et al.* 2005; Spana *et al.* 2020). This could suggest that *Yippee* directly or indirectly affects the biosynthetic pathway between dopamine and NBAD, as has been demonstrated for *black*, *ebony*, and *tan* (Wittkopp *et al.* 2003; Phillips *et al.* 2005; True *et al.* 2005; Yamamoto and Seto 2014; Massey and Wittkopp 2016).

Yippee protein was first isolated in a protein-trap screen for *Drosophila* proteins that could interact with Hemolin, a moth immunoglobulin (Roxström-Lindquist and Faye 2001). Hemolin shares some sequence identity with the *Drosophila* protein Neuroglian, which affects the fly immune response (Williams 2009). Taken together, this could indicate a role for *Yippee* in *Drosophila* immunity, but *Yippee* mRNA expression did not appear to be upregulated upon activation of the immune response (Roxström-Lindquist and Faye 2001).

Yippee protein shares high sequence identity with the mouse and human YPEL (Yippee-like) family of conserved proteins: 43.4–48.5% identity with YPEL1-YPEL4, and most notably, 70.8% identity with YPEL5, a component of the E3 ubiquitin ligase complex (Hosono *et al.* 2004; Lampert *et al.* 2018). Yippee is a hydrophilic protein with no signal peptide at the N-terminus, so it was initially hypothesized to be an intracellular protein (Roxström-Lindquist and Faye 2001). In support of this hypothesis, immunocytochemistry showed that YPEL5 localizes to the nuclei of COS-7 (monkey kidney fibroblast-like) cells, and Yippee, YPEL5, and almost all other known YPEL proteins share a putative nuclear localization sequence of (K/R)YKEG(K/R) (Hosono *et al.* 2004, 2010). Further, Yippee and most every other identified YPEL protein has a zinc-finger-protein-like sequence of 2 pairs of cysteines spaced apart by 52 amino acids (C-x_2_-C-x_52_-C-x_2_-C). C-x_2_-C is a common motif used by metallothioneins and other metal-sensing proteins to bind zinc, copper, and other metal ions (e.g. Buchman *et al.* 1989; Koch *et al.* 1997; Egli *et al.* 2006). Therefore, the Yippee C-x_2_-C-x_52_-C-x_2_-C domain may form a metal-binding pocket (Roxström-Lindquist and Faye 2001).

The high sequence similarity between Yippee and YPEL5 is intriguing, owing to a web of connections between YPEL family proteins, the E3 ubiquitin ligase complex, copper homeostasis, and adult cuticle melanization: (1) Copper ions act as cofactors for several enzymes in the *Drosophila* melanization pathway, specifically the intracellular enzymes tyrosine hydroxylase and dopamine monooxygenase, which synthesize dopamine from L-tyrosine; and laccase, a secreted enzyme that converts secreted dopamine to dopamine quinone (True *et al.* 2005; Dittmer and Kanost 2010; Riedel *et al.* 2011; Armstrong *et al.* 2013; Yamamoto and Seto 2014; Massey and Wittkopp 2016; Spana *et al.* 2020). (2) Copper is required in the *Drosophila* ectoderm for adult cuticle melanization, and excessive copper import into ectodermal cells causes hyperpigmentation, possibly by increasing the activity of melanization enzymes (Zhou *et al.* 2003; Norgate *et al.* 2006; Turski and Thiele 2007; Binks *et al.* 2010; Armstrong *et al.* 2013; Vasquez-Procopio *et al.* 2020; Zhang *et al.* 2021). (3) The *Drosophila* E3 ubiquitin ligase complex regulates copper homeostasis in the ectoderm at least in part by regulating expression, degradation, and/or intracellular localization of the copper transporters Ctr1A and ATP7 (Zhang *et al.* 2020, 2021). (4) In a similar fashion, the mammalian E3 ubiquitin ligase complex also regulates copper homeostasis (Mufti *et al.* 2007; Brady *et al.* 2010). (5) YPEL5, a component of the mammalian E3 ubiquitin ligase complex, shares high sequence identity with Yippee (Hosono *et al.* 2004; Lampert *et al.* 2018), and their shared sequence includes the putative copper-binding domain. (6) *pnr* > *Yippee*RNAi darkens cuticle along the dorsal midline of the thorax (Fig. 3, a, d, and e); this indicates that *Yippee* acts in ectodermal cells—the same cells in which copper homeostasis affects pigmentation—to regulate body color. This broad but circumstantial evidence suggests a scenario where Yippee negatively regulates copper levels in ectoderm cells, perhaps via the E3 ubiquitin ligase complex. Under this model, loss of *Yippee* function would be expected to increase intracellular copper levels, darkening the cuticle. Future experiments could directly test this hypothesis.

### Investigating the cell nonautonomy of *sable*

Lewis (1955) observed gynandromorphs that were mosaic for *s^+^* and *s* and concluded that the *sable* body color is cell nonautonomous, i.e. *s*^+^ cells can rescue the phenotype of *s* cells within the same fly. Our tissue-specific RNAi experiments lend further insight into Lewis’ observations, suggesting that cells with loss of *Yippee* function can only be rescued by nearby cells within the same tissue:

First, we found no evidence that loss of *Yippee* function in the ectoderm can be rescued by wild-type *Yippee* function in other tissues. *pnr* > *Yippee*RNAi phenocopied *sable* body color along the dorsal midline of the thoracic cuticle, while *elav*, *fkh*, and r4 > *Yippee*RNAi did not noticeably darken color on the scutum or anywhere else on the body (Fig. 3). *pnr*-GAL4 is a dorsal ectoderm-specific driver, suggesting that *Yippee* acts in the ectoderm to affect body color. The negative results for the *elav*, *fkh*, and r4 drivers suggest that *Yippee* expression in postmitotic neurons, salivary glands, or fat body does not affect body color from a distance (Chintapalli *et al.* 2007; Brown *et al.* 2014; and see Supplementary Materials and Methods > I for GAL4 driver references). This does not rule out long-distance action completely; a more exhaustive screen of GAL4 drivers would be required to determine if *Yippee* acts in any tissue other than the ectoderm to regulate cuticle pigmentation.

Second, a comparison of *nub* > *Yippee*RNAi and *pnr > Yippee*RNAi results suggests that *sable* nonautonomy has a limited range within the wing disk. *nub*-GAL4 and *pnr*-GAL4 are expressed in adjacent ectodermal cells in the wing disk with similar timing, *nub*-GAL4 in the prospective wing blade domain, and *pnr*-GAL4 in the prospective notum (Heitzler *et al.* 1996; Azpiazu and Morata 2000; Calleja *et al.* 2000). However, *nub* > *Yippee*RNAi and *pnr > Yippee*RNAi effects did not appear to overlap: *nub* > *Yippee*RNAi curved wings with complete penetrance, even though scutal cuticle color was normal. Conversely, *pnr* > *Yippee*RNAi darkened small patches of cuticle on the dorsal thorax even though the wings were not curved (Fig. 3; Table 1). While these findings do not refute Lewis’ hypothesis of cell nonautonomy, they do suggest that *Yippee* function in the prospective wing blade does not influence the phenotype of the prospective notum and vice versa.

Third, we observed evidence of *Yippee* nonautonomy in our *pnr* > *Yippee*RNAi experiments. If *Yippee* had been a cell autonomous trait, and assuming that *Yippee* is expressed in the ectoderm of the anterior thorax with similar timing to *pnr*-GAL4, we might have expected to see *pnr* > *Yippee*RNAi affect body color along a broad stripe extending anteriorly to the head, as was seen with treatments such as *pnr*-GAL4 driving *CG1673*RNAi, *ebony*RNAi, or RNAi of the E3 ubiquitin ligase gene *Vhl* (our observations; Mummery-Widmer *et al*. 2009; Massey *et al.* 2019a; Zhang *et al.* 2021). However, we found that *pnr*-GAL4 driving either *Yippee*RNAi construct (V 46977, V 39899) only darkened small patches on the midline of the scutellum and posterior scutum (Fig. 3e). This domain did not expand significantly if flies were raised at 29°C to increase GAL4 function and with UAS-*Dcr2* to increase the RNAi effect (BL 25758; data not shown). The sizes of these dark patches were consistent with those seen in *pnr* > *Yippee*RNAi images presented on the IMBA Bristle Screen Database (Mummery-Widmer *et al*. 2009). In summary, the *pnr* > *Yippee*RNAi pigmentation pattern was narrower than expected based on the width of the *pnr*-GAL4 expression domain, suggesting that wild-type Yippee protein effects extend somewhat into the *pnr* > *Yippee*RNAi stripe: nonautonomous, but short range.

Our data are consistent with Lewis’ hypothesis of *sable* cell nonautonomy, but might suggest that *Yippee*^+^ cells can only rescue nearby *Yippee*^−^ cells within the same tissue. These results are reminiscent of mosaic analysis of another well-known body color gene: In gynandromorphs mosaic for *yellow*, *yellow*^+^ cuticle rescued immediately adjacent *yellow* cuticle (Hannah 1953). *yellow* encodes an L-dopachrome isomerase that affects melanin synthesis; this enzyme has a signal peptide that directs transport to the ER, glycosylation, and subsequent secretion (Drapeau 2003; Hinaux *et al.* 2018). It therefore makes intuitive sense that *yellow* exhibits cell nonautonomy. In contrast, the Yippee protein does not have a signal peptide, and so it has been hypothesized to be intracellular (Roxström-Lindquist and Faye 2001). It is possible that Yippee regulates a downstream signal that is secreted or otherwise relayed to other cells; for example, laccase and its substrate dopamine are secreted in the melanization pathway, so Yippee might affect this process (True *et al.* 2005; Yamamoto and Seto 2014; Massey and Wittkopp 2016; Spana *et al.* 2020). Further investigation of *Yippee* nonautonomy, such as replication of Lewis’ results using mitotic clonal analysis, seems merited (Germani *et al.* 2018).

### How the *Yippee^sable1^* mdg4 insertion might affect *Yippee* function?

The *s^1^* strain has an mdg4 retrotransposon element inserted in the 5′-UTR of *Yippee* (Fig. 5a; GenBank accession # OM135585). Such an insertion could affect *Yippee* transcript levels and/or structure, and our analysis of *Yippee* mRNA is consistent with both hypotheses: (1) RT-qPCR showed a roughly 80% reduction of *Yippee* cDNA levels in *s^1^* mutants vs *s^+^* controls (Fig. 5j). (2) All of the *s^1^ Yippee* cDNAs that we cloned and sequenced contained residual mdg4 sequences in the 5′-UTR and were also missing a segment of the 5′-UTR due to splicing (Fig. 6). Most of these altered 5′-UTRs carry upstream ORFs. Any of these mdg4-related alterations could be the cause of the *Yippee^sable1^* phenotype by altering mRNA transcription rate, stability, export, and/or translation rate. The observed temperature sensitivity of *Yippee^sable1^* remains unexplained. One hypothesis is that *Yippee^sable1^* transcript levels and/or splicing may also be temperature sensitive. Alternatively, a downstream or independent element in the pigmentation pathway might itself be temperature sensitive, but it only manifests a phenotype in the presence of reduced Yippee protein levels.

It is likely that at least some *Yippee^sable1^* transcripts were translated into functional Yippee protein, for 2 reasons. First, the observed cDNAs carry a functional *Yippee* ORF. Second, the *Yippee^sable1^* phenotype is hypomorphic: the phenotype is enhanced over Df(1)Exel6245 (Fig. 2) and not as severe as the *Yippee^Chi-A^* and *Yippee^Δ1^* strong loss-of-function phenotypes (Fig. 5), suggesting some residual function despite the mutated 5′-UTR and lower transcript level.

The established genetic interaction between *Yippee^sable1^* and *suppressor of sable* [*su(s)*] may provide a foothold for further research of *Yippee^sable1^* transcript functionality. Ironically, the mechanism by which *suppressor of sable* mutations suppress *sable* is unclear, as molecular studies of *su(s)* have focused on its genetic interactions with genes that had already been cloned, such as *vermillion*. Su(s) protein is thought to be a component of transposon defense machinery: it binds pre-mRNA that contains TE insertion sequence near the 5′ end of the transcript, and there is evidence that this interaction negatively affects transcription rate while targeting transcript for degradation by the exosome. Mutations in *su(s)* suppress certain TE insertion mutations in the 5′-UTR of other loci by reducing degradation of TE-containing pre-mRNA, allowing for retention, splicing, and nuclear export of more transcript. After splicing and export, it is thought that at least some of the salvaged aberrant mRNA is translated into functional protein, rescuing the phenotype of the suppressed mutation (Searles and Voelker 1986; Rutledge *et al.* 1988; Searles *et al.* 1990; Geyer *et al.* 1991; Murray *et al.* 1997; Kuan *et al.* 2004, 2009). Now that *Yippee* has been identified as the *sable* gene, the molecular basis of the genetic interaction between *su(s)* and *Yippee^sable1^* can be investigated.

### *CG1673* also affects body color

As described in our *Results*, RNAi and mutant alleles of *CG1673* lightened cuticle color somewhat (our observations; Mummery-Widmer *et al*. 2009); IMBA Bristle Screen Database). *CG1673* encodes a predicted branch-chain-amino-acid transaminase, which is involved in leucine and valine biosynthesis (Maguire *et al.* 2015). We did not investigate *CG1673* further, but it may be of interest to researchers of body pigmentation.

### Delayed developmental rate

*Yippee^Chi-A^*, *Act5C* > *Yippee*RNAi, and r4 > *Yippee*RNAi flies all exhibited delayed development of at least 2 days from egg to adult, without any particular stage clearly presenting as a rate-limiting step. In addition, *Yippee^Δ1^* mutants exhibited developmental delay to the pharate adult stage. While not surprising, this phenotype had not been reported for *sable* mutants before. r4 is a fat body-specific driver (Lee and Park 2004), *Yippee* is expressed at high levels in the fat body (Chintapalli *et al.* 2007; Brown *et al.* 2014), and r4 > *Yippee*RNAi was the only tissue-specific RNAi treatment that we tested that produced a noticeable developmental delay. Therefore, *Yippee* may act in the fat body to affect developmental rate.

### How *Yippee* might affect bristles?

We found evidence that *Yippee* also affects scutellar, OC, and PV bristle numbers (Table 1). Bristle counts are particularly sensitive to a variety of environmental factors such as teratogens and oxidative stress, making false-positive “mutant” phenotypes possible (Barik and Mishra 2019; Priyadarsini *et al.* 2019). However, our negative controls rarely if ever displayed abnormal bristle counts, and each *Yippee* bristle phenotype was independently verified with multiple genotypes (Table 1): Ectopic scutellar bristles were seen in all 3 *Yippee* mutant lines, with *s^1^* failing to complement *Yippee^Chi-A^* and *Yippee^Δ1^*. A copy of UAS-*Yippee* rescued *s^1^* ectopic bristles, and *Act5C* > *Yippee*RNAi and *pnr* > *Yippee*RNAi both phenocopied the mutant trait. In contrast, the missing OC/PV bristle trait did not behave as a straightforward loss-of-function phenotype; among all the genotypes examined for our body color investigation, only *s^1^*/Df(1)Exel6245 and *pnr* > *Yippee*RNAi flies were missing OC/PV bristles at significant frequencies. Among the tissue-specific *Yippee*RNAi treatments that we tested, *pnr* > *Yippee*RNAi was the only one that phenocopied the *Yippee* bristle traits, suggesting that *Yippee* expression in the ectoderm affects bristle development.

Beyond its apparent ectoderm specificity, how *Yippee* affects bristle development remains an open question. The bristle sensory organ is produced through multiple rounds of cell division and cell fate determination. At the start of metamorphosis, an array of proneural clusters (PNCs) is established in the developing ectoderm. A single sensory organ precursor (SOP) is selected within each PNC, and this SOP divides asymmetrically to produce pIIa and pIIb. Subsequently, pIIa divides to generate the socket and bristle cells, and pIIb divides to produce the neuron and sheath cells; these 4 descendants of the SOP comprise the bristle sensory organ (Schweisguth *et al.* 1996; Gomez-Skarmeta *et al.* 2003; Furman and Bukharina 2008; Schweisguth 2015).

Although disruption of any of the above steps could cause ectopic or missing bristles, the positioning of ectopic bristles may hint at the mechanism underlying the *Yippee* mutant phenotype. A mutation that causes ectopic PNCs would be capable of producing ectopic bristles that are well-separated from neighboring bristles. On the other hand, a mutation that acts downstream of PNC formation would be expected to produce ectopic bristles that are always adjacent to or adjoining a neighboring bristle, because both bristles originated from the same PNC and/or SOP (Usui *et al.* 2008; Karbowniczek *et al.* 2010; Troost *et al.* 2015; Court *et al.* 2017). At least to some extent, our analysis of *Yippee* mutants and *Yippee*RNAi supported both scenarios: (1) In some cases, ectopic scutellar bristles were well-separated from neighboring bristles (Figs. 3e and 5e; Supplementary Results > Supplementary Fig. S7e). However, (2) the majority of ectopic scutellar bristles were closely associated with a neighbor, even though these bristles always had their own separate socket (Figs. 2c, 2h, 3c, 4d, 5d, 5e, and 5g; Supplementary Results > Supplementary Fig. S7c). (3) Missing OC/PV bristles were only seen in *s^1^*/Df(1)Exel6245 and *pnr* > *Yippee*RNAi flies at significant frequencies (Table 1; Fig. 3e; Supplementary Results > Supplementary Fig. S7e). Taken together, *Yippee* may have some effect on initial PNC formation (1, 3), but more consistently, our observations suggest a role for *Yippee* at or downstream of SOP formation (2).

Activation of the Notch EGF-like protein by its ligand Delta control several cell fate decisions in the SOP lineage: In the SOP, Notch signaling inhibits adjacent PNC cells from forming ectopic SOPs. Thereafter, Notch specifies pIIa cell fate, then socket and sheath cell fates as pIIa and pIIb divide (Furman and Bukharina 2008; Schweisguth 2015; Sjoqvist and Andersson 2019). Interestingly, some *Notch* and *Delta* mutations phenocopy loss of *Yippee* function. Certain mutations in *Notch* and *Delta* cause scutellar bristle duplication or loss, and mutations in the *Abruptex* domain of *Notch* are especially effective at causing loss of OC and PV bristles (Lindsley and Zimm 1992). While *Notch* itself has not been strongly associated with body pigmentation, several *Delta* mutations have been associated with dark body color (Schultz 1929; Cramer 1980; Lindsley and Zimm 1992). With these parallels in mind, it is worth considering whether *Yippee* interacts with Notch/Delta signaling. As discussed above (*The Biochemical and Physiological Role of Yippee Remains Unclear*), Yippee shares high sequence similarity with YPEL5, a member of the mammalian E3 ubiquitin ligase complex (Hosono *et al.* 2004; Lampert *et al.* 2018), and the E3 ubiquitin ligases Neuralized, Mindbomb, and Deltex affect *Drosophila* bristle formation by regulating the function, endocytosis, and degradation of Notch and Delta (Dietrich and Campos-Ortega 1984; Lindsley and Zimm 1992; Wang and Struhl 2005; Miller and Posakony 2018; Dutta *et al.* 2021). Taken together, it is possible that Yippee interacts with the *Drosophila* E3 ubiquitin ligase complex to regulate Notch/Delta signaling in the developing bristle organ.

### How *Yippee* might affect wing morphology, adult emergence, and cuticle composition?

The new *Yippee* alleles, as well as some *Yippee*RNAi treatments, appeared to disrupt *Yippee* function more severely than *Yippee^sable1^*, revealing novel phenotypes such as curved wings and failed adult emergence. Defining tissue/cell specificity is a helpful foothold to investigate gene function. We have this foothold with the *Yippee* wing phenotype: The *nub*-GAL4 driver primarily expresses in the prospective wing blade, an ectodermal tissue (Azpiazu and Morata 2000), and *nub* > *Yippee*RNAi flies phenocopied the curved wings of *Act5C* > *Yippee*RNAi and *Yippee^Chi-A^* flies (Figs. 3, h and  and 5e; Table 1). Therefore, it is likely that wing morphology depends at least in part on *Yippee* expression within the developing wing ectoderm. The wing is an efficient and sensitive gauge in genetic interaction studies (e.g. Dean *et al.* 2015; Ellis *et al.* 2015; Straub *et al.* 2020), so the *Yippee* wing phenotype could be a useful starting point to research the mechanisms of *Yippee* function throughout the fly.

It is unclear whether *Yippee* affects adult emergence directly or indirectly. *Yippee^Δ1^* pharate adults arrested at P13-14 and did not initiate emergence. In contrast, *Act5C* > *Yippee*RNAi and *Yippee^Chi-A^* pharate adults at least attempted to emerge, but often became stuck while exiting the operculum. *Yippee^sable1^* did not show obvious emergence problems. One hypothesis for the difference in severity is that *Yippee^Δ1^* is a molecular null allele, so it might be expected to cause a more severe phenotype than *Yippee^Chi-A^* and *Yippee^sable1^*, which express some *Yippee* transcript (Fig. 5). Our experiments do not rule out the possibility that *Yippee^Δ1^* contains a second-site mutation that causes lethality at an earlier stage in addition to the emergence problems seen with the *Yippee*RNAi and *Yippee^Chi-A^* genotypes. Taken together, these observations suggest that *Yippee* is required for either (1) initiation and/or execution of the emergence behavior or (2) viability at P13-14, shortly before adult emergence can begin. Unlike the body pigment, wing, developmental rate, and bristle phenotypes, the tissue specificity of the *Yippee* adult emergence phenotype is unknown—indeed, it is possible that this phenotype is not tissue-specific: strong loss of *Yippee* function might have a generally adverse effect on, for example, metabolism or immunity, leading to lethality at the sensitive transition to the adult stage. Future experiments could test whether *Yippee* affects adult emergence and viability by its expression in any particular tissue.

Finally, given *Yippee’*s role in cuticle pigmentation, it may be worth investigating whether it affects other cuticle biomaterial properties. The processes of pigmentation (melanization) and hardening of the adult cuticle (sclerotization) are biochemically related, both depending on the copper-dependent dopamine monooxidase (laccase) encoded by *straw* (Suderman *et al.* 2006; Flaven-Pouchon *et al.* 2020; Spana *et al.* 2020). Therefore, it is possible that *Yippee* regulates sclerotization as well as melanization. Further study could determine if and how exoskeleton composition, crosslinkage, and hardness are affected by *Yippee* (Jacobs 1985; Flaven-Pouchon *et al.* 2016, 2020).

### Concluding remarks

The authors initially, through our roles as educators and students, undertook this study as a guided inquiry within the classroom. Investigating a 110-year-old question that arose from work by some of the first *Drosophila* researchers reinforced the relevance of studying classical genetics and, perhaps more importantly, engaged students in the discovery process. The identification of *Yippee* as the *sable* gene, its compelling connections to a variety of threads in the literature, and the “*Yippee* toolkit” provide many opportunities for the research community to investigate a diverse—yet potentially interrelated—array of topics including melanin biosynthesis, the E3 ubiquitin ligase pathway, copper homeostasis, wing development, RNA surveillance, bristle formation, Notch/Delta signaling, and adult emergence.

## Data availability

The Supplementary Materials and Methods, Supplementary Data, and Supplementary Results have been deposited on the GSA figshare portal: https://doi.org/10.25387/g3.19059725. The *Yippee^Chi-A^* and *Yippee^Δ1^* mutant strains are currently available from the authors and have been deposited at the Bloomington Stock Center (BL 93858 and BL 93859). *Yippee* sequences from *s^1^* have been deposited in GenBank under accession number OM135585. The sequences of the *Yippee^Chi-A^* and *Yippee^Δ1^* mutations are described in the Supplementary Materials and Methods > IV. *s^1^* pupal and adult *Yippee* cDNA sequences are in the Supplementary Results. *Drosophila* modular cloning toolkit (Dmo) parts are available from the authors on request. Raw scutal gray value data and *P*-values for pairwise comparisons (Tukey’s HSD) are provided in an XLSX file (Supplementary Data). Table 1 of this manuscript essentially presents raw bristle and wing data.
